# Supplementary material for: Tunable anti-ambipolar vertical bilayer organic electrochemical transistor enable neuromorphic retinal pathway
Source: Nat Commun. 2024 Jul 26;15:6309. doi: 10.1038/s41467-024-50496-6 (PMC11282299; doi:10.1038/s41467-024-50496-6)
Supplement: Supplementary file 1 — Supplementary Information [file 41467_2024_50496_MOESM1_ESM.docx]

**Supporting Information**

**Tunable Anti-Ambipolar Vertical Bilayer Organic Electrochemical Transistor enable Neuromorphic Retinal Pathway**

*Zachary Laswick^1‡^, Xihu Wu^2‡^, Abhijith Surrendran^1^, Zhong-Liang Zhou^2^, Xudong Ji^1^, Giovanni Maria Matrone^1^*, Wei Lin Leong^2^*, Jonathan Rivnay^1,3^**

^‡These authors contributed equally to this work^

^1^ Department of Biomedical Engineering, Northwestern University, Evanston, IL, USA

^2^ School of Electrical and Electronic Engineering, Nanyang Technological University, 50 Nanyang Avenue, Singapore 639798, Singapore.

^3^ Department of Materials Science and Engineering, Northwestern University, Evanston, IL, 60208, USA

E-mail: [giovanni.matrone@northwestern.edu](mailto:giovanni.matrone@northwestern.edu), [wlleong@ntu.edu.sg](mailto:wlleong@ntu.edu.sg), [jrivnay@northwestern.edu](mailto:jrivnay@northwestern.edu)

Keywords: oects, vertical, spiking, neurons, bilayer, logic, antiambipolar


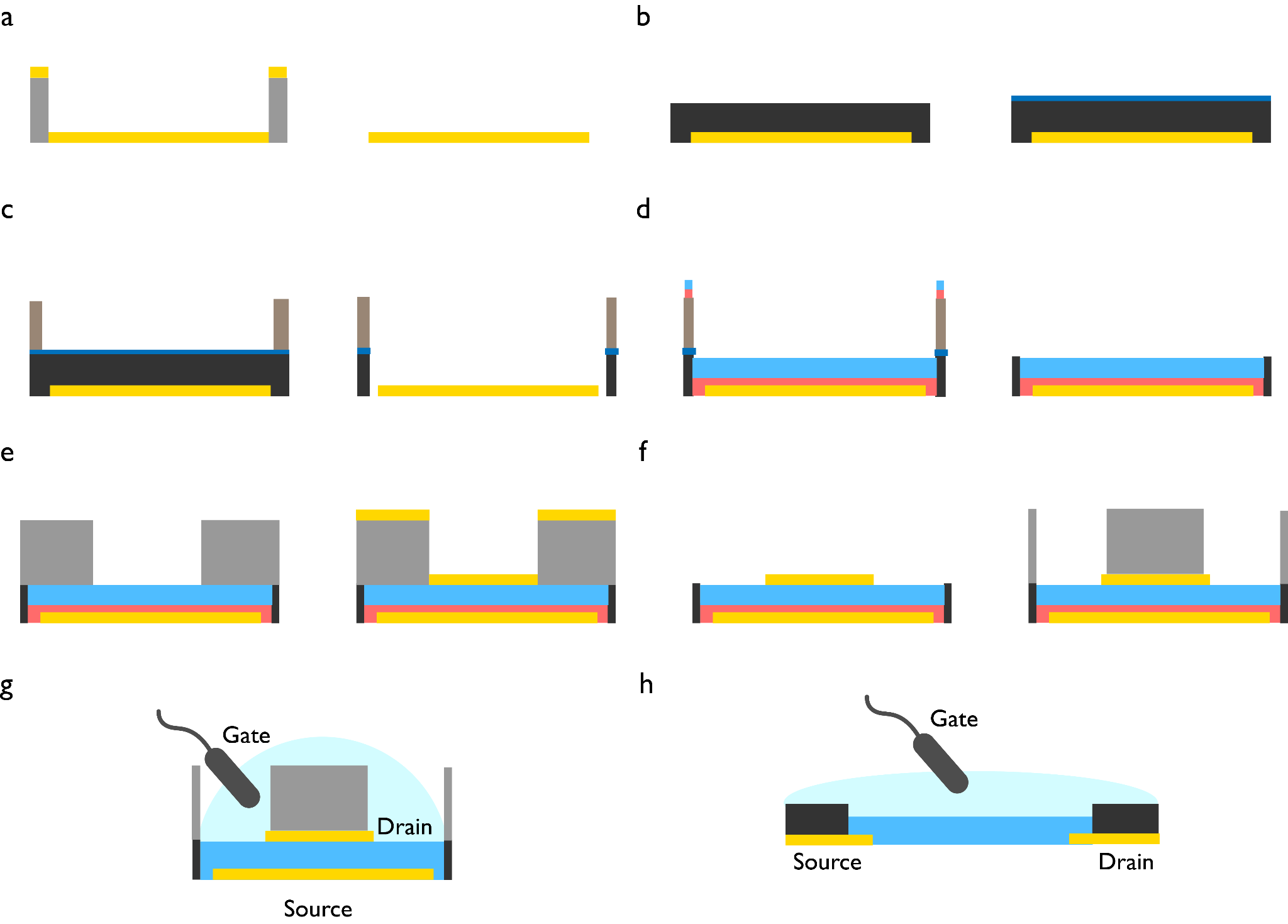


**Figure S1: Bilayer vOECT Device Fabrication Procedure a,** Patterning of the bottom electrode (gold) via standard photolithography methods (light gray).**b,** Deposition of parylene C layer (dark gray) with subsequent soap (dark blue) for peel-off patterning of vOECT channels. **c,** Patterning of SU8 (beige) layer and etching of underlying parylene C layers (dark gray) for peel-off patterning. **d,** Spin coating of the channel materials via one material then the next (red for BBL and blue for PEDOT, as an example). This step is followed by peel-off patterning of the channel materials. **e,** Patterning of the top electrode (gold) via standard photolithography methods (light gray). **f,** Patterning of top passivating layer via negative photoresist (light gray) patterning. **g,** Schematic of a vOECT architecture, using the aforementioned color coding, with labeled Gate, Drain, and Source electrodes. **h,** Schematic of a conventional lateral (coplanar) architecture, using the aforementioned color coding, with labeled Gate, Drain, and Source electrodes.


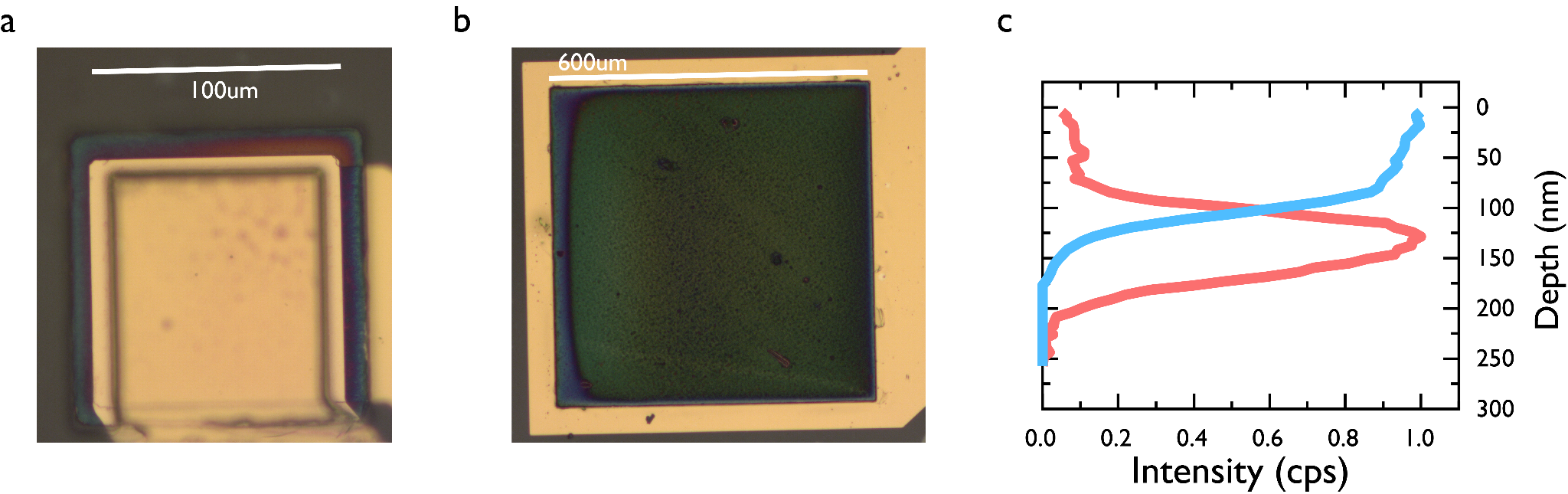


**Figure S2: BBL/PEDOT microscopy and bilayer XPS characterization a,** Microscopy image of a *W*=100 µm BBL/PEDOT bilayer vOECT at 100x magnification. **b,** Microscopy image of a BBL/PEDOT bilayer film at 10x magnification. **c,** XPS data from a BBL/PEDOT bilayer film on glass, where Blue is the sulfur signal and Red is the nitrogen signal.


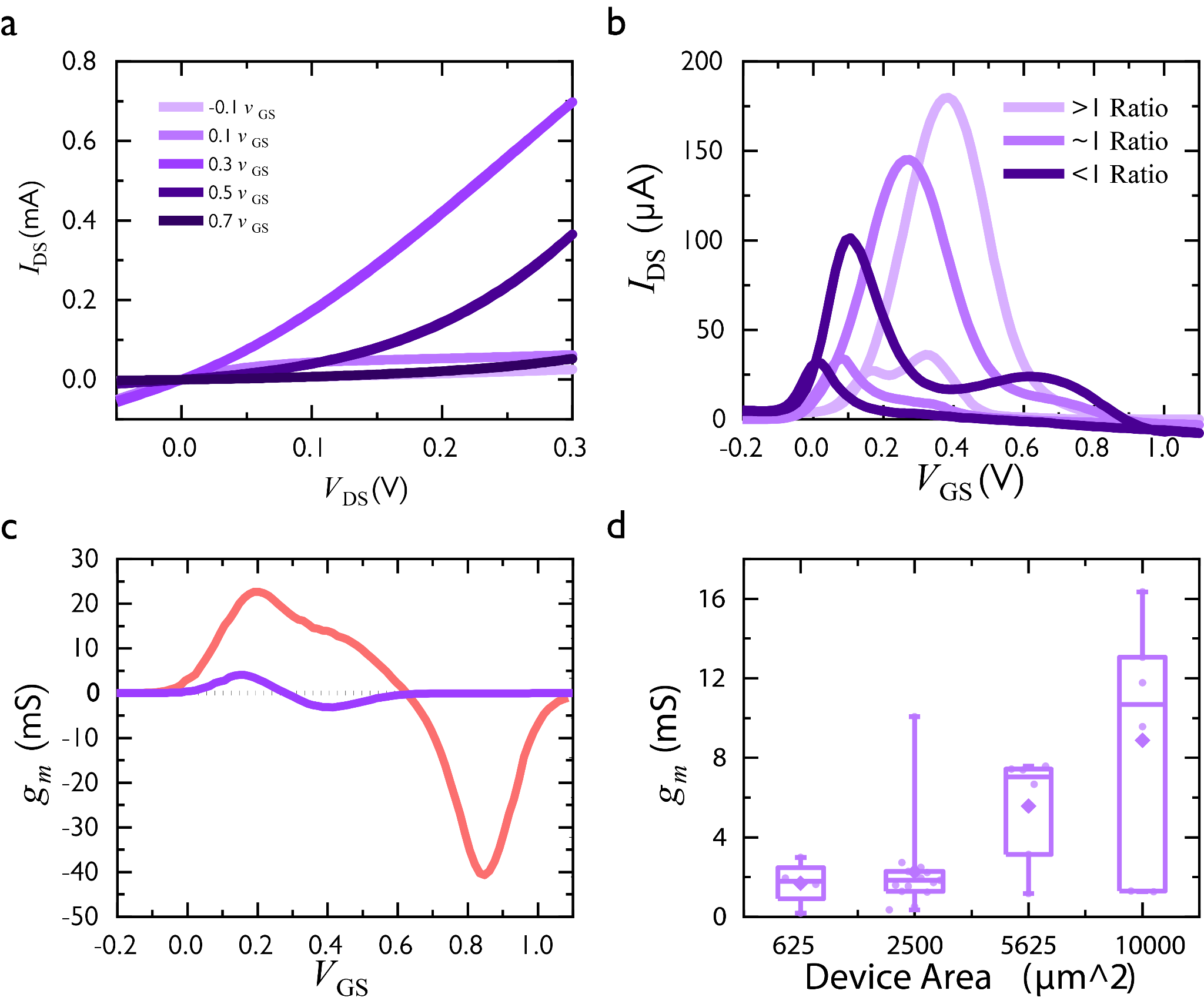


**Figure S3: BBL/PEDOT Output, transfer, and channel length modulation characteristics. a,** Output curve of a BBL/PEDOT bilayer vOECT (*W*=100 µm). **b,** Example devices for changing the channel length ratio (ie the length of PEDOT layer/length of BBL layer) of the BBL/PEDOT bilayer vOECT as discussed in Section 2 and **Figure 1** (*W*=75 µm, *V*_DS_ =0.15V). **c,** Example transconductance of a BBL/PEDOT bilayer vOECT (purple) and a BBL vOECT (red) (*W*=100 µm, *V*_DS_=0.1V). **d,** Change in peak transconductance as a function of device area (*W*x*W*) (*V*_DS_=0.15V, n>4, mean (diamond), median (line), quartiles (box), and 5-95 whiskers displayed, p=0.0001).


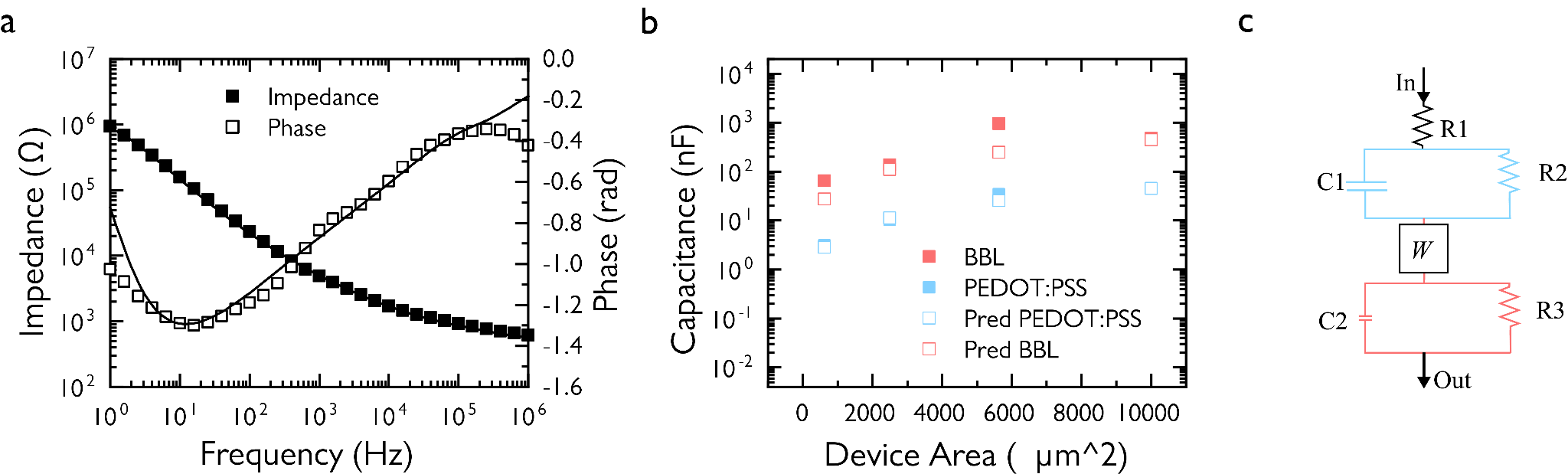


**Figure S4: EIS of BBL/PEDOT bilayer vOECTs** **a,** EIS data from the BBL/PEDOT bilayer vOECT (*V*_offset_= 0.3, *W*=50 µm) with fitting, **b,** Internal Capacitance scaling of each layer with device area based on EIS data fitting (closed squares) or reported volumetric capacitance values (open squares)(n=1)[^1^](https://www.zotero.org/google-docs/?ul3det) **c,** Circuit used to fit the EIS data, consisting of two Randles cells (blue for the PEDOT:PSS layer and red for the BBL layer) separated by a Warburg element.


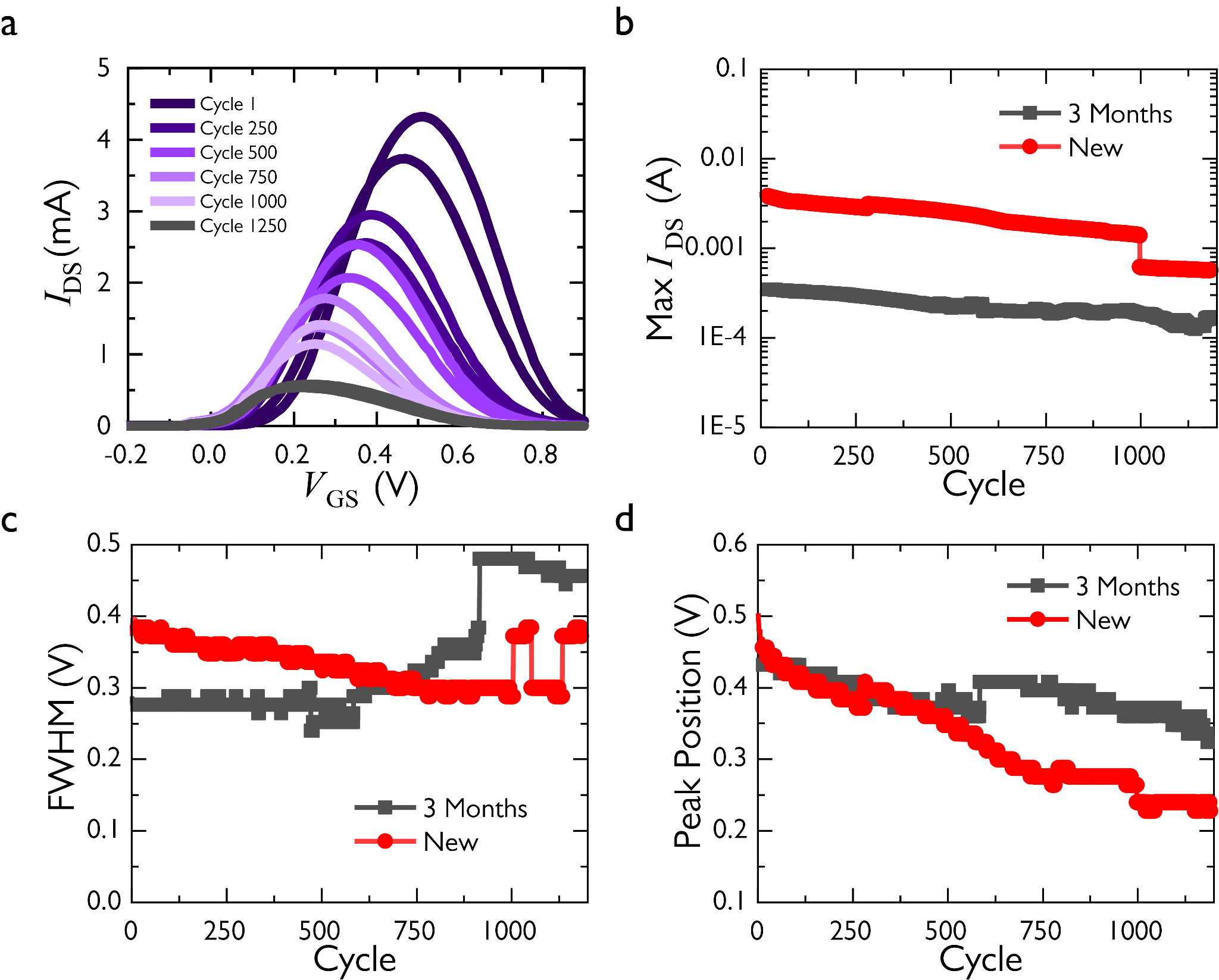


**Figure. S5: Stability of the BBL/PEDOT bilayer vOECT a,** Transfer Curve measurements from a BBL/PEDOT bilayer vOECT (*W*=100 µm, *V*_DS_=0.15 V) from different cycle numbers. **b,** Stability of peak current in the BBL/PEDOT bilayer vOECT over cycle number. **c,** Stability of FWHM in the BBL/PEDOT bilayer vOECT over cycle number. **d,** Stability of peak position in the BBL/PEDOT bilayer vOECT over cycle number.


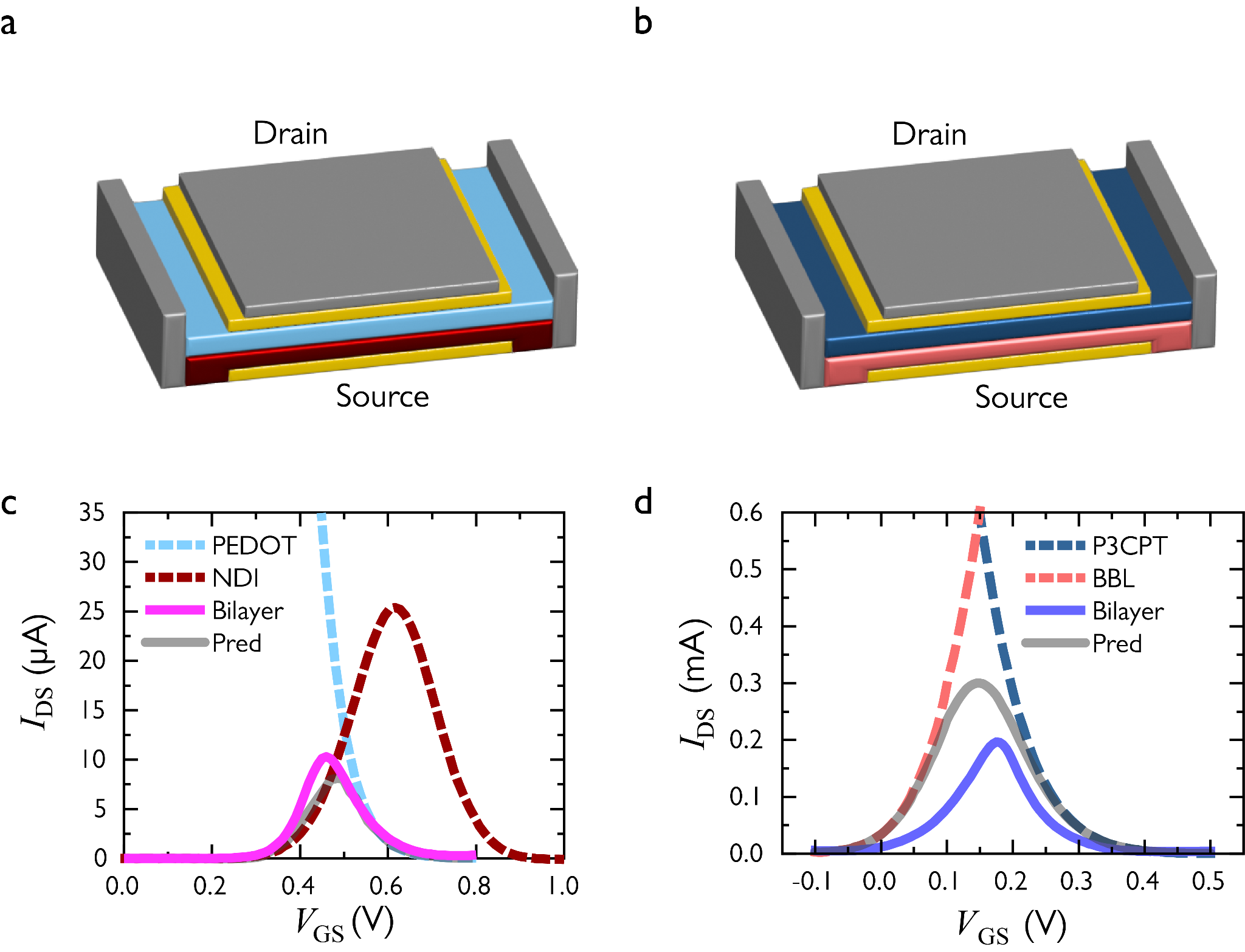


**Figure S6: In-series resistance deconvolution and prediction of alternative material bilayer vOECTs a,** Device diagram for the NDI/PEDOT bilayer vOECTs with corresponding NDI layer on bottom and PEDOT layer on top of the NDI layer. **b,** Device diagram for the BBL/P3CPT bilayer vOECT with corresponding BBL layer on bottom and P3CPT layer on top of the BBL layer. **c,** Deconvolution of the NDI/PEDOT bilayer vOECT (solid magenta) with corresponding prediction (gray) (*W*=100 µm, *V*_DS_=0.15 V). **d,** Deconvolution of the BBL/P3CPT bilayer vOECT (solid blue) with corresponding prediction (gray)(*W*=100 µm, *V*_DS_=0.3V).


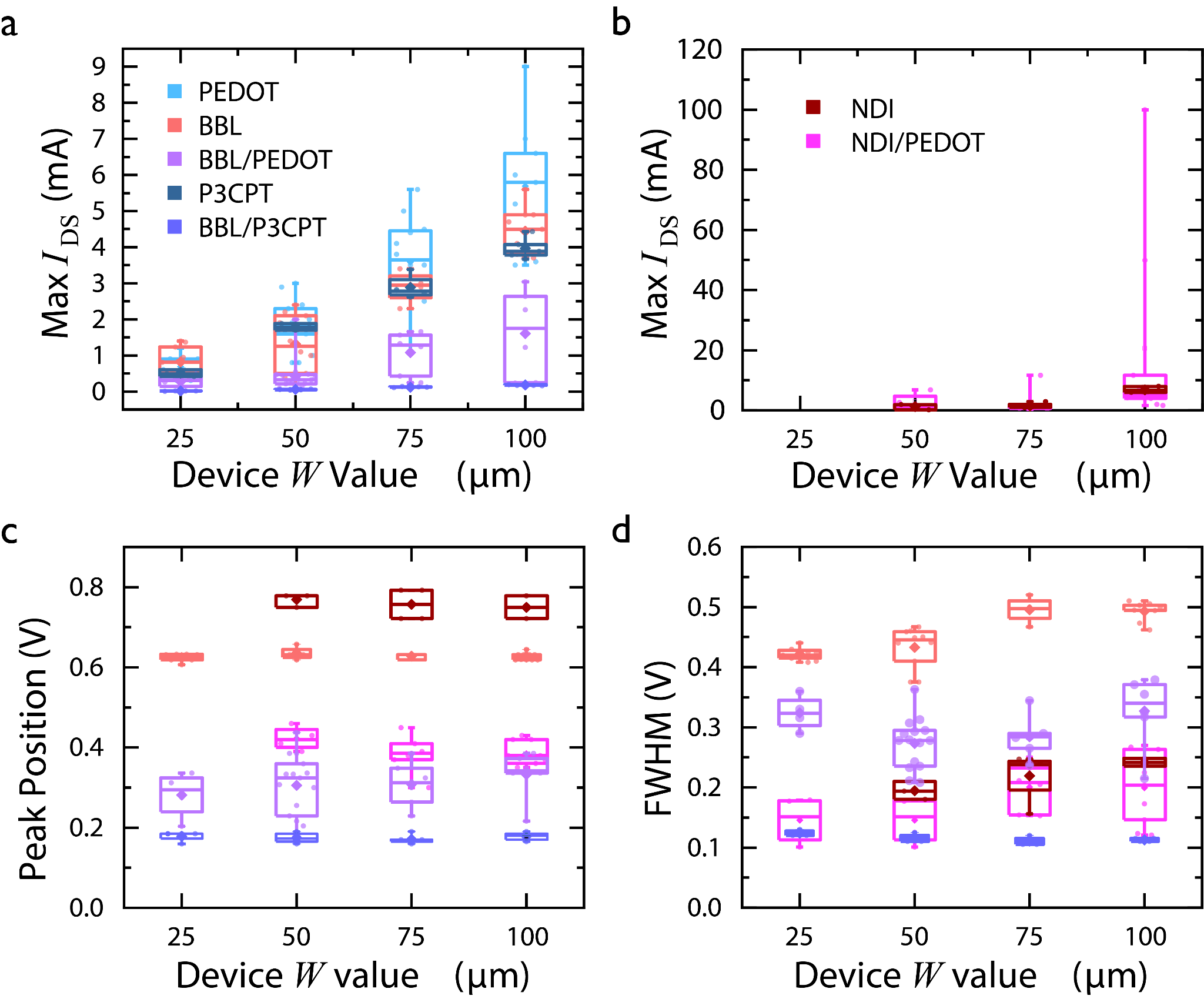


**Figure S7: Influence of W-dimension on max current, peak position, and FWHM for vOECTs. a,** Max *I*_DS_ for vOECT devices within the 0.01-10mA range as *W* value increases (n>3). **b,** Max *I*_DS_ for vOECT devices within the 0-60µA range as *W* value increases (n>3).**c,** Shift in peak position as device *W* value increases (*V*_DS_ =0.15V)(n>2). **d,** Shift in FWHM as device *W* value increases (*V*_DS_=0.15V)(n>2). In all panels, the mean (diamond), median (line), quartiles (box), and 5-95 whiskers are displayed.


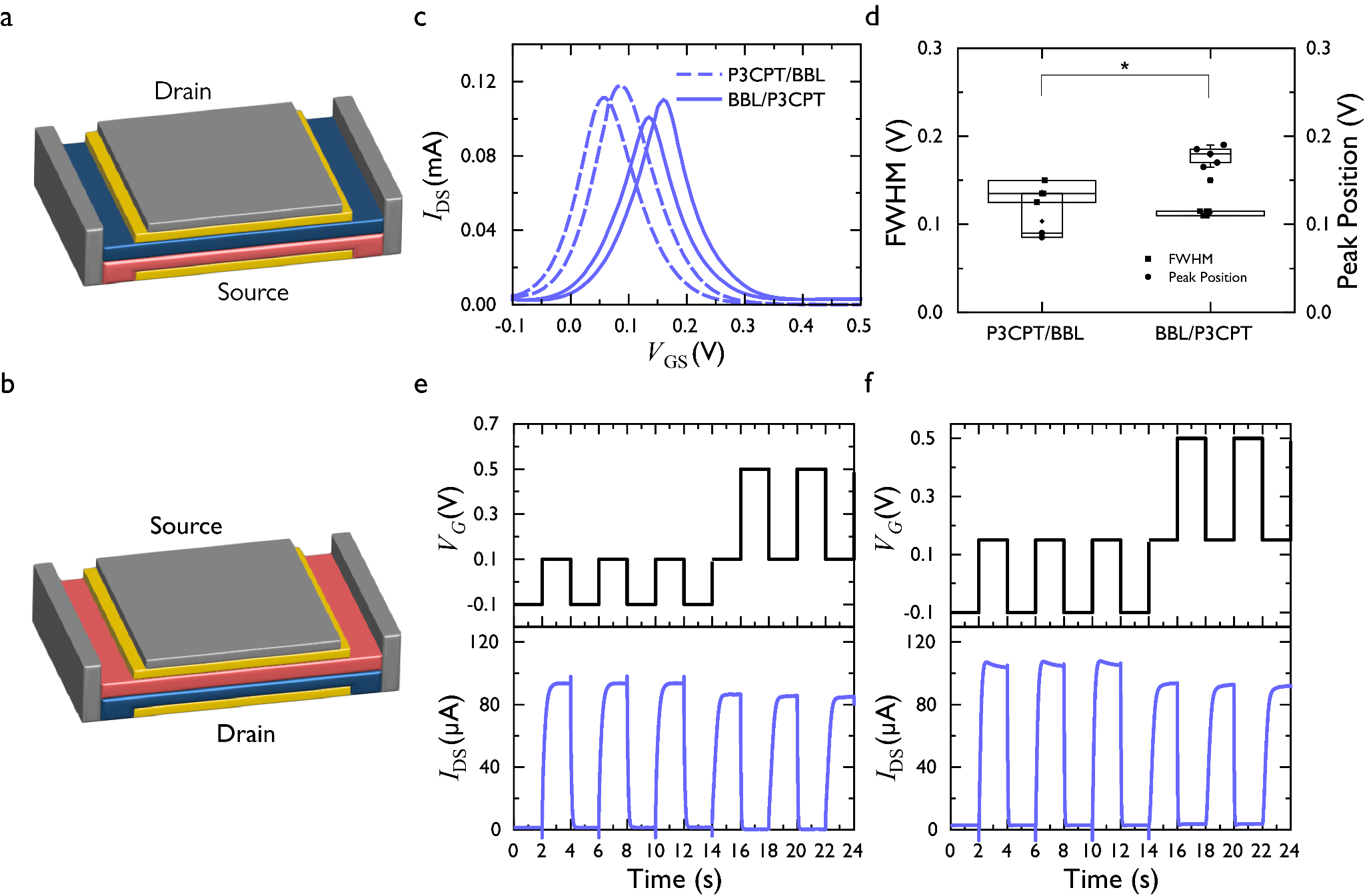


**Figure S8: Effects of N-type on top and P-type on bottom a,** Device diagram for the BBL/P3CPT bilayer vOECTs with corresponding BBL layer on bottom and P3CPT layer on top of the BBL layer. **b,** Device diagram for the P3CPT/BBL bilayer vOECT with corresponding P3CPT layer on bottom and BBL layer on top of the P3CPT layer. **c,** Corresponding transfer curves of bilayer devices with the layers swapped in both increasing and decreasing V_GS_ conditions to demonstrate hysteresis. In both devices the increasing V_GS_ curve reached a higher peak current than the decreasing V_GS_ curve (*W*=100 µm, *V*_DS_=0.3V). **d,** Corresponding Peak Position (circle) and FWHM (square) differences between bilayers. (P3CPT/BBL n=3, BBL/P3CPT n=5, *W*=100 µm, *V*_DS_=0.3V, p=0.0053 for FWHM, p=0.00129 for Peak Position, mean and standard deviation displayed) **e,** Pulsing behavior of the P3CPT/BBL bilayer with response times of 181 and 245 ms depending on the V_GS_ range (*W*=100 µm, *V*_DS_=0.3V). **f,** Pulsing behavior of the BBL/P3CPT bilayer with response times of 113 and 218 ms depending on the *V*_GS_ range (*W*=100 µm, *V*_DS_=0.3V).

**SI Note 1:**

**Figure S1** shows the fabrication procedure for the bilayer vOECTs consisting of standard photolithography, peel-off, and spin coating steps to create a gold - n-type - p-type - gold structure. For reference, microscopy images of the BBL/PEDOT bilayer devices are provided, while XPS was performed on a bilayer film deposited on a glass substrate to reduce interference from gold electrodes and better define the stacked films structure **(Figure S2)**. The bilayer is clearly formed with a minimal intermixing layer (10-20 nm), with thickness similar to those of individual films coated on glass substrates **(Figure S2)**. This narrow intermix area may improve ion intercalation, promoting the penetration of ions through PEDOT:PSS into BBL films, but as demonstrated in Figure 1e, Figure S3, and Figure S4 this phenomenon has minimal impact on device characteristics in comparison to the bulk properties of each layer. Furthermore an example output curve and transconductance curve are provided for the BBL/PEDOT bilayer vOECTs **(Figure S3)**. It is of interest to note that the transconductance of the BBL/PEDOT bilayer vOECTs is significantly more symmetrical than the transconductance of BBL, and that the transconductance of the BBL/PEDOT bilayer vOECT scales appropriately with device W-value, which is provided as the area value in **Figure S3**[^2^](https://www.zotero.org/google-docs/?8upeaL). Additionally, example devices from different channel lengths are provided **(Figure S3b)**, which are obtained by changing the thickness of the PEDOT:PSS component of the bilayer. This results in layers with higher and lower p-type resistances which allow a shift (to higher and lower voltages) the anti-ambipolar peak of the bilayer device **(Figure S3)**. Across these tests, the W-values were varied from 25x25, 50x50, 75x75, to 100x100 µm, while the length of the PEDOT channel varied from 75 nm to 110 nm to 300 nm (**Figure 1f-g, Figure S3**).

Electrochemical Impedance Spectroscopy (EIS) was also conducted on each device dimension to characterize the internal capacitance of the bilayer vOECTs for circuit applications. Using the bottom electrode as the working electrode, the devices were tested with offset voltages spanning the active range device. To model the circuit of the bilayer device, a standard Randle’s Cell is used for each layer with a Warburg element in between to mimic the ion-diffusion limitations imposed by the interface between the two materials. For reference, an example fit of the Bode plot data can be seen in **Figure S4**. The dominant capacitance of these devices is represented by the BBL layer, likely due to this material’s high *C**[^1–3^](https://www.zotero.org/google-docs/?lYy3To). Furthermore, these values provide an order of magnitude estimate of the internal capacitance of each layer, which are used in circuit modeling of the bilayer and their circuit applications within SI Notes 2 and 3.

The stability of the bilayer OECT is necessary to enable its application in neuromorphic and bioelectronics applications, thus the BBL/PEDOT bilayer vOECT operations have been tested up to 1200 cycles, immediately after fabrication and in the extreme case of devices stored in ambient conditions for three months **(Figure S5)**. Reduction of the bilayer OECT electronic performance is mainly related to the PEDOT:PSS layer’s degradation. Indeed, a reduced peak conductance and a shift to lower peak position voltages with increasing cycle number is observed, which is compatible with an increase of resistance caused by the degradation of the single layer PEDOT:PSS OECTs. This component failure is further evidenced by the shift of the negative transconductance region of the bilayer anti-ambipolar curve over cycles, still consistent with a potential degradation of the PEDOT:PSS layer **(Figure S5)**. Furthermore, as this layer fails and swelling leads to increased contact resistance, the ON/OFF ratio of the device decreases until the FWHM becomes unstable, at which point the device is no longer usable for the applications demonstrated. As such, the stability of the bilayer vOECT is dependent on the application requirements, where the applications demonstrated do not require high stability in peak position but high stability in FWHM. Alternatively, a gaussian probabilistic network application would require minimal variation in peak position and FWHM over cycle count, and thus this system would need improvement in its stability to be a viable option. Improvements in bilayer vOECT stability can consist of alternative p-type material selection and the usage of flexible top electrode materials to reduce the degradation caused by PEDOT:PSS and eliminate the possibility of a swelling-induced increase of the contact resistance[^1,3–6^](https://www.zotero.org/google-docs/?T6AMSO).

Following the in-series procedure used to rationalize the bilayer vOECT transfer curves, similar circuit predictions and overlays applied to the case of NDI/PEDOT and BBL/P3CPT bilayers further confirmed the approach. As such, in-series predictions can be generalized to these alternative materials to define the resulting bilayer transfer curve **(Figure S6)**. Additionally, these materials and their bilayers were tested at various W-values (25x25, 50x50, 75x75, 100x100 µm), over which the peak current of each device scaled according to device area **(Figure S7)**. Focusing on the BBL, P3CPT, and BBL/P3CPT devices, the change in peak current over area were incredibly similar for the BBL and P3CPT, which resulted in bilayer having no difference in peak position and FWHM over device area **(Figure S7)**. Furthermore, the change in peak current over area was not different enough between material combinations to result in an meaningful effect on peak position or FWHM for any resulting bilayer except BBL/PEDOT, which exhibited a slight relationship, but statistically significant (p<0.05), between peak position and device area **(Figure S7)**. This dependance is consistent with the in-series predictions as the resistance of the PEDOT:PSS component decreased more than the BBL component as W value increased leading to an increase in the peak position, similar to the effect seen by changing channel length ratio, but less drastic. Although the NDI and PEDOT devices exhibited orders of magnitude differences in peak current, the interaction point occurs near the threshold voltage of PEDOT and therefore the change in current with W-value is minimal, resulting in no impact of W-value on peak position or FHWM **(Figure S7)**. Thus, the device dimension engineering can provide a mechanistic approach for fine tuning of a bilayer vOECT’s peak position, if specific material properties are desired but the resulting peak position is unfavorable.

The use of alternative material combinations also enabled probing of the layer order dependence of anti-ambipolar bilayer vOECT characteristics, such as peak position, peak current, FWHM, and response time. Primarily, P3CPT does not acid crystalize, (like PEDOT:PSS) in the presence of MSA, and it does not dissolve in this solvent, thereby it can be used as a p-type material beneath the n-type material **(Figure S8)**. As seen in **Figure S8**, this mildly affects the peak position and FWHM. However, the peak current of the resulting bilayer vOECT device changes as a result of the hindered ion transport to the P3CPT layer and the promoted ion transport into the BBL layer leading to a slight shift in each layer's threshold voltage. Altered ion transport is further evidenced by the increased response time of the BBL on top bilayers (P3CPT/BBL) compared to the BBL/P3CPT bilayers, which results in less favorable devices for many circuit applications. For example, the increased response time of this reversed stack would lead to slower switching between logic states in the case of anti-ambipolar logic circuits, or lower spiking frequencies in the case of anti-ambipolar driven spiking circuits.


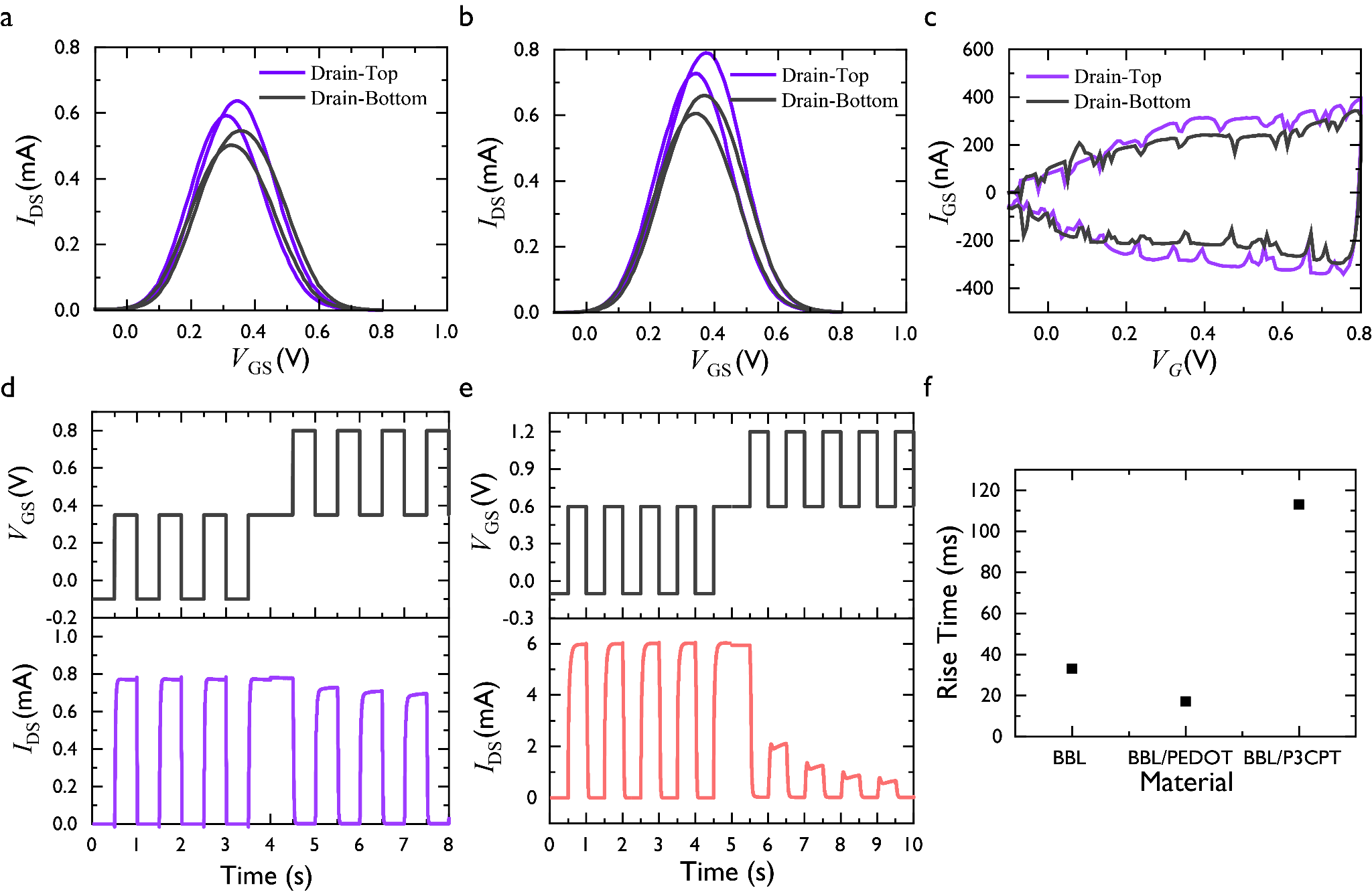


**Figure S9: Devices used in BBL/PEDOT Logic Gates** **a,** Transfer curves of the device used to create the Logic gates in **Figure 3** (*W*=75 µm, *V*_DS_=0.1). **b,** Transfer curves of the device used to create the Logic gates in **Figure 3** (*W*=75 µm, *V*_DS_=0.1). **c,** Leakage current of the device in **Figure S9 a,b**. **d,** Pulsing behavior of the device in **Figure S9 b**.**e,** Pulsing behavior of a BBL vOECT (*W*=75 µm, *V*_DS_=0.1). **f,** Rise time of different BBL-based bilayers (n-1).


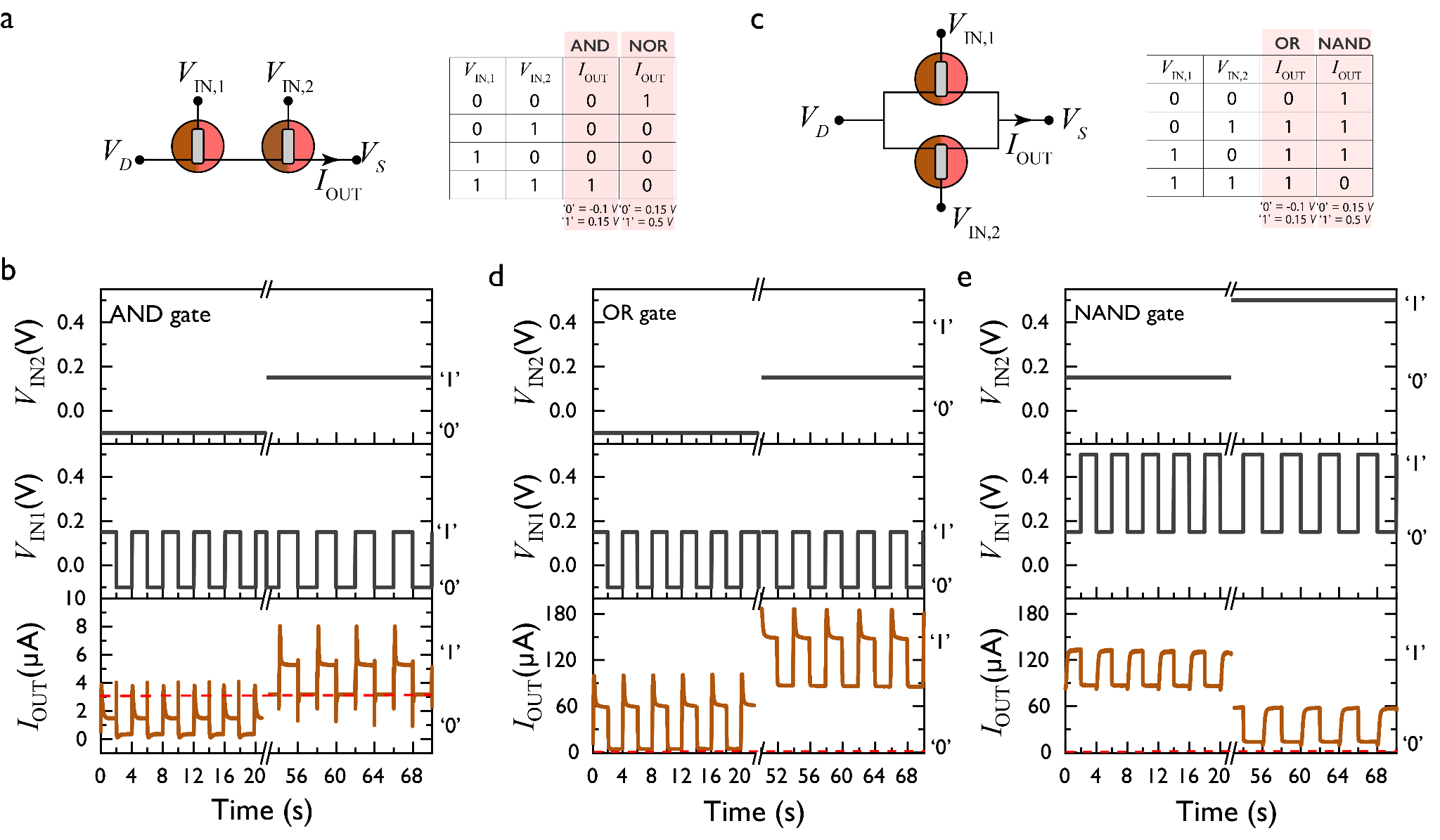


**Figure S10: Logic Gates created using alternative bilayer vOECTs.** **a,** Circuit design for AND/NOR gate. **b,** Input voltage and output current from a BBL/P3CPT AND gate. **c,** Circuit design for OR/NAND gate. **d,** Input voltage and output current from a BBL/P3CPT OR gate. **e,** Input voltage and output current from a BBL/P3CPT NAND gate.


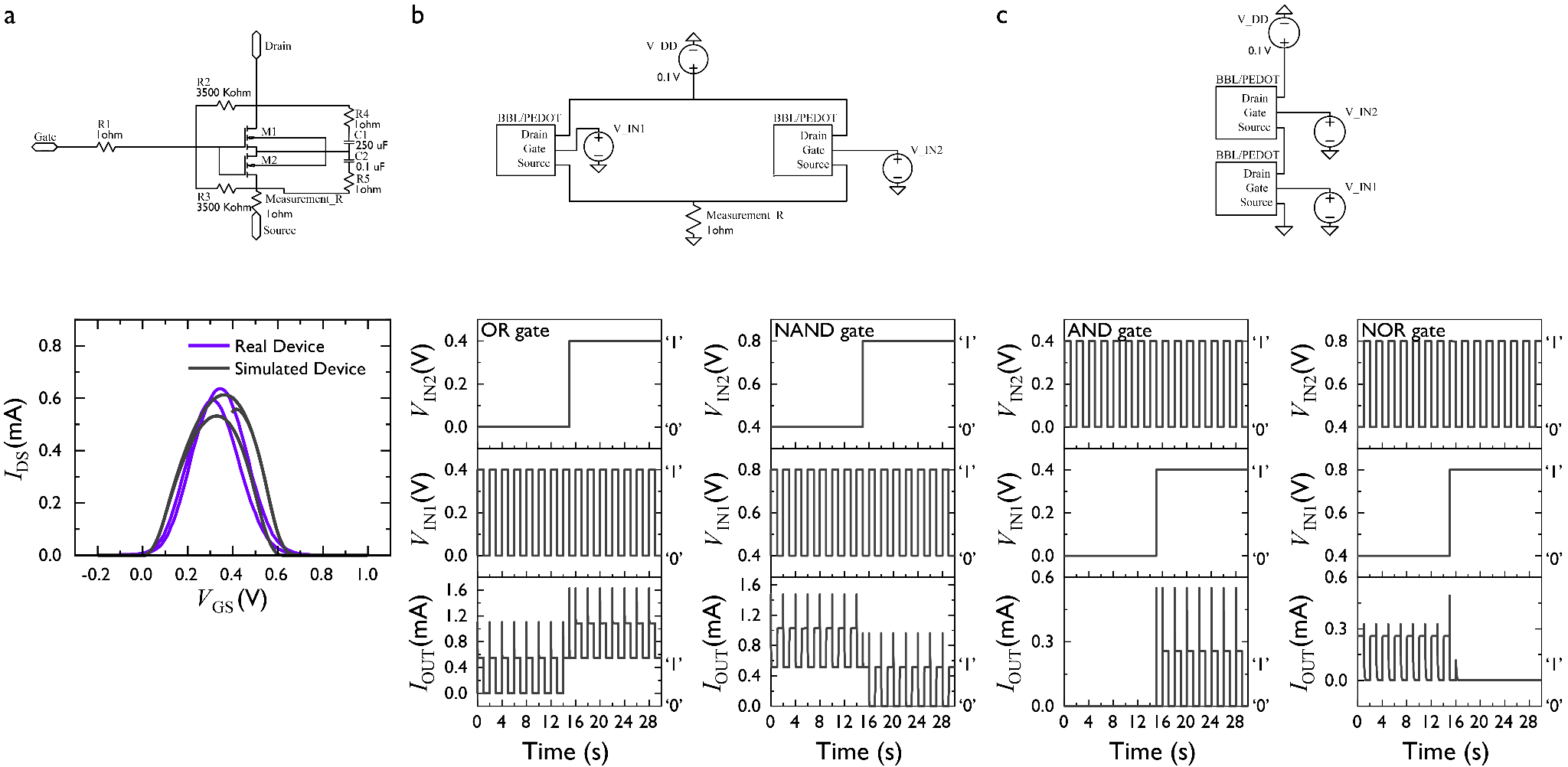


**Figure S11: LTSpice simulation of the BBL/PEDOT bilayer vOECT and corresponding Logic Circuits a,** LTSpice circuit of an anti-ambipolar bilayer device and the corresponding transfer curve comparison. **b,** Simulated OR/NAND gate with results. **c,** Simulated AND/NOR gate with results.


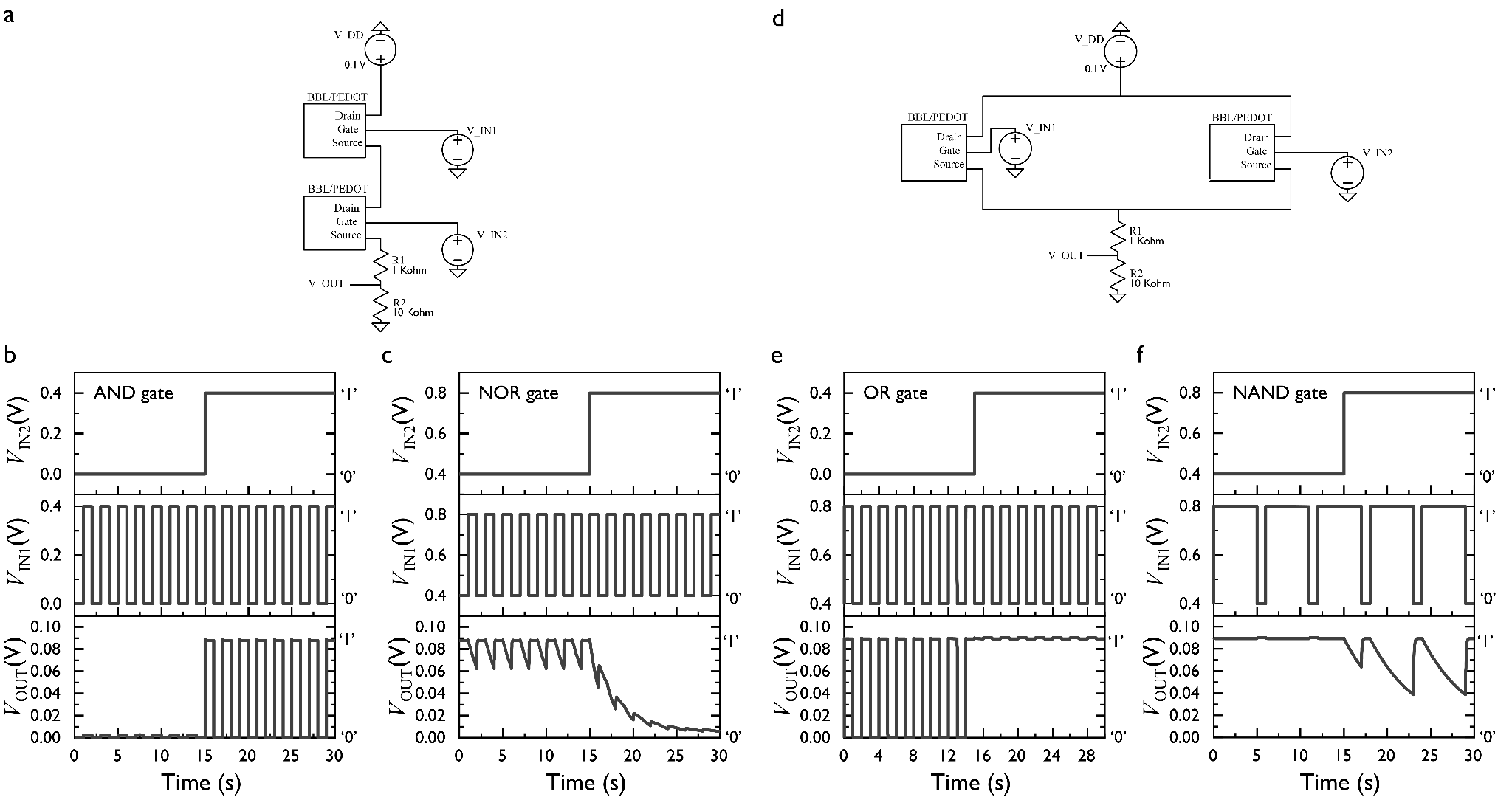


**Figure S12: Addition of a Voltage Divider to the Logic Circuits a,** Circuit design for AND/NOR gate with voltage divider. **b,** Input and output voltage from a simulated AND gate. **c,** Input and output voltage from a simulated NOR gate. **d,** Circuit design for OR/NAND gate with voltage divider. **e,** Input and output voltage from a simulated OR gate. **f,** Input and output voltage from a simulated NAND gate.


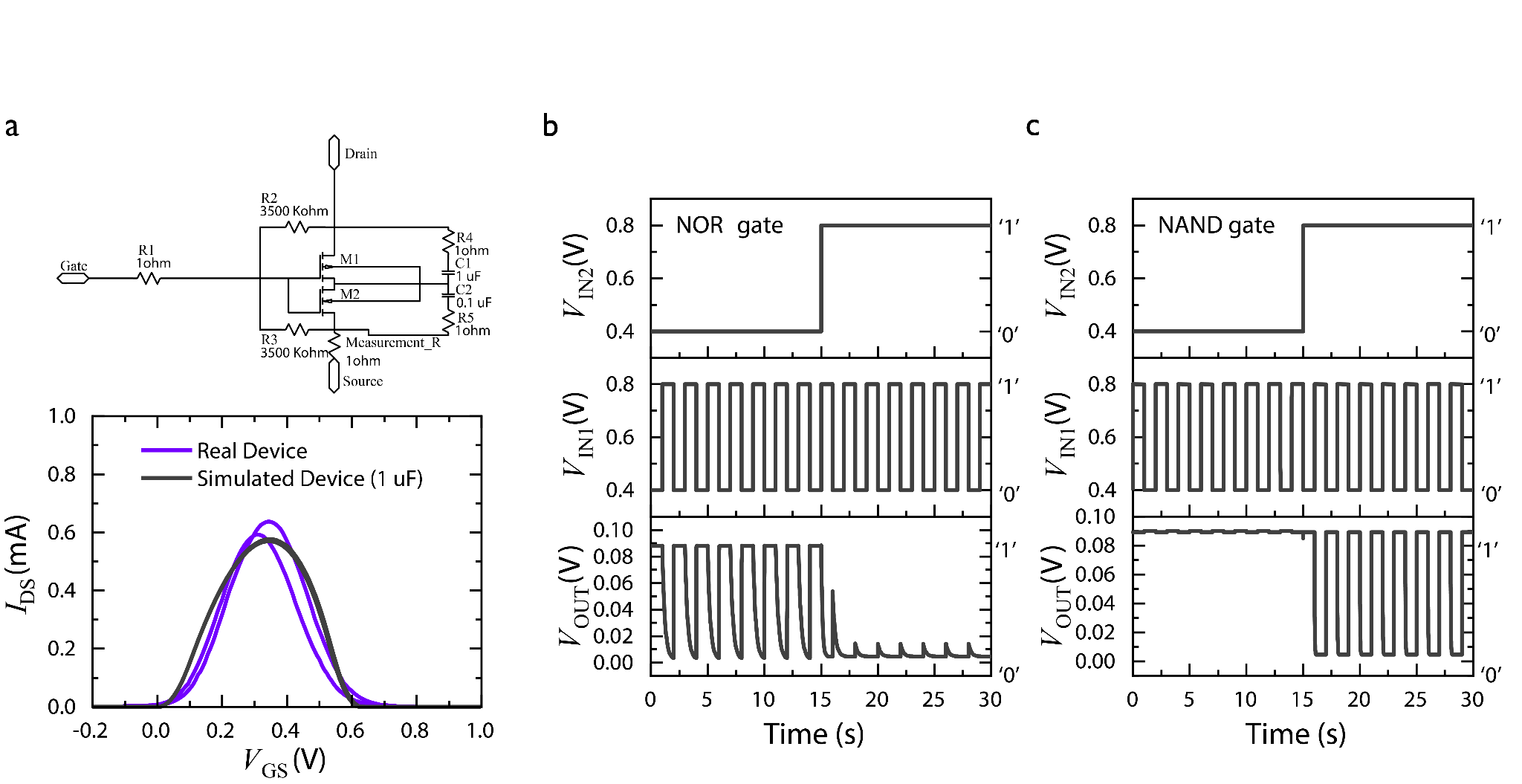


**Figure S13: Reduction of Device Capacitance to Measured Values Improves NOR and NAND Gates a,** LTSpice circuit of an anti-ambipolar bilayer device with the reduced internal capacitance and the corresponding transfer curve comparison. **b,** Simulated NOR gate and voltage divider using reduced internal capacitance. **c,** Simulated NAND gate and voltage divider using reduced internal capacitance.

**SI Note 2:**

Primarily, BBL-based bilayers were investigated in circuit applications due to their increased stability and high on/off ratio, as seen with the devices used for the Logic Gates in **Figure S9**. Specifically, BBL-PEDOT was chosen due to its fast rise time and stable ON-OFF ratio, compared to the other bilayer materials **(Figure S6, Figure S9)**. However, changing the p-type material does allow for lower voltages for the True and False states, further highlighting the benefits of the tunable bilayer anti-ambipolar vOECT. For example, logic gates with BBL/P3CPT devices **(Figure S10)** show reduced *V*_GS_ but the inferior ON/OFF ratio of the BBL/P3CPT leads to decreased performance **(Figure S10)**. Using the device presented in **Figure S9** as a model, an LTSpice circuit was developed to model the bilayer behavior **(Figure S11a)**. The model consists of a p-type and n-type transistors in series along with resistors and capacitors to simulate the leakage and hysteresis of the bilayer device. The simulated logic gates closely resemble the results presented in **Figure 3**, enabling exploration of approaches to improve the circuit performance **(Figure S11)**. For example, **Figure S12** demonstrates the conversion of the output current to an output voltage for use in other logic gates via the addition of a voltage divider to the output **(Figure S12)**. However, the use of high internal capacitances in the model to match the hysteresis of the actual device leads to slowed responses for the ON to OFF based logic gates **(Figure S12c,f)**. By reducing the internal capacitance of the model to the order of magnitude of device values, the discharge time of the circuits is improved, but the simulated hysteresis fails to match device values **(Figure S13)**. Therefore, the addition of a voltage divider can convert the current output of the logic devices into a voltage output for applications in traditional large-scale logic circuits. However, a current-based output is also desirable for neuromorphic applications allowing, for example, control over the current-input into diverse spiking circuits, such as a leaky integrate and fire circuit neuron model or an HH-circuit neuron model[^2,7–10^](https://www.zotero.org/google-docs/?EQCCvX).


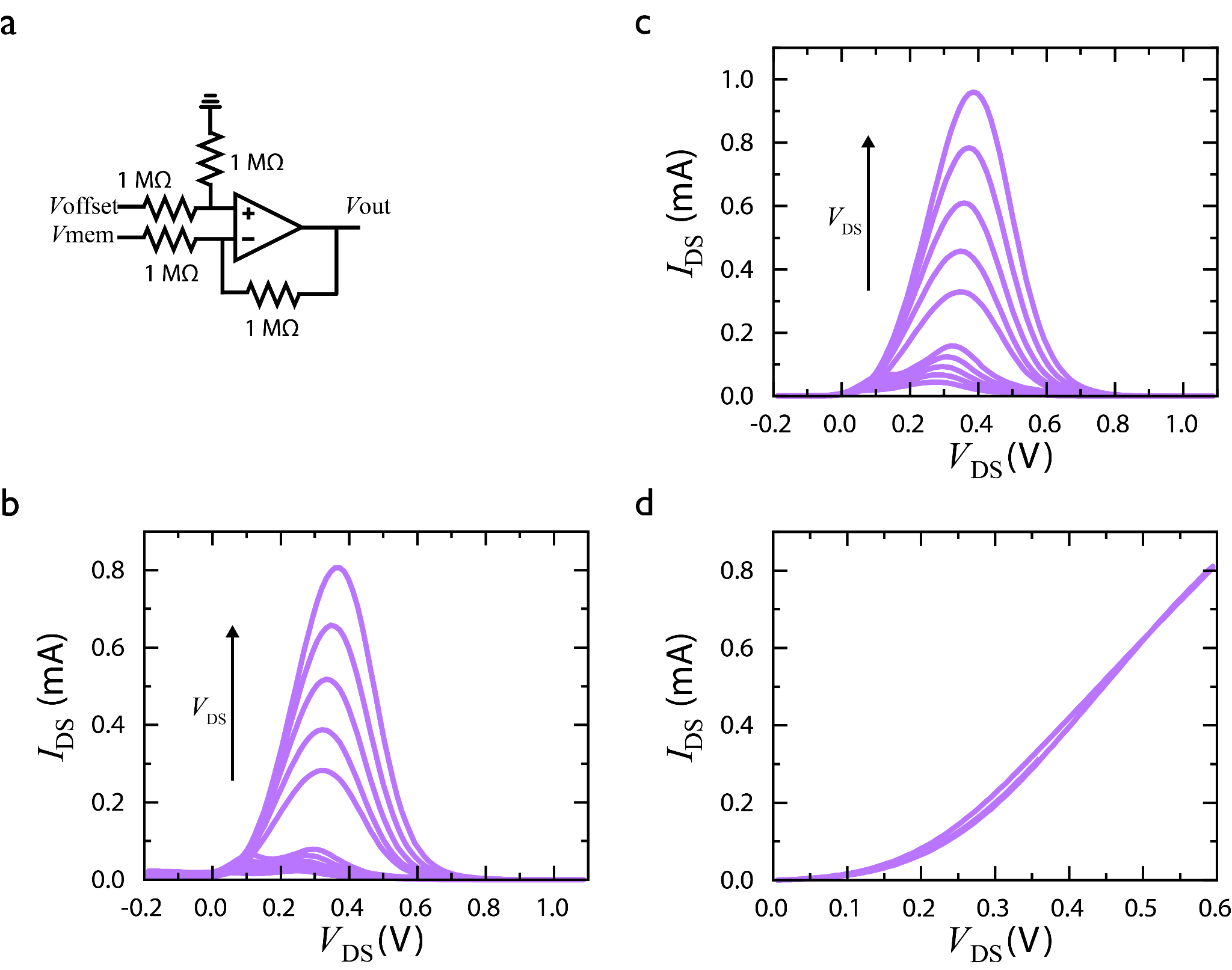


**Figure S14: Spiking circuit devices a,** Circuit design for the differential operational amplifier. **b,** Transfer curves of the BBL/PEDOT vOECT used for the Na Ion Channel (*V*_DS_ = 0.05, 0.075, 0.1, 0.125, 0.15 V, *W*=50 µm). **c,** Transfer curves of the BBL/PEDOT vOECT used for the K Ion Channel (*V*_DS_ = 0.05, 0.075, 0.1, 0.125, 0.15 V, *W*=50 µm). **d,** Sweep of the K Ion Channel device when *V*_GS_ and *V*_DS_ are connected by a 1 Mega-ohm resistor.


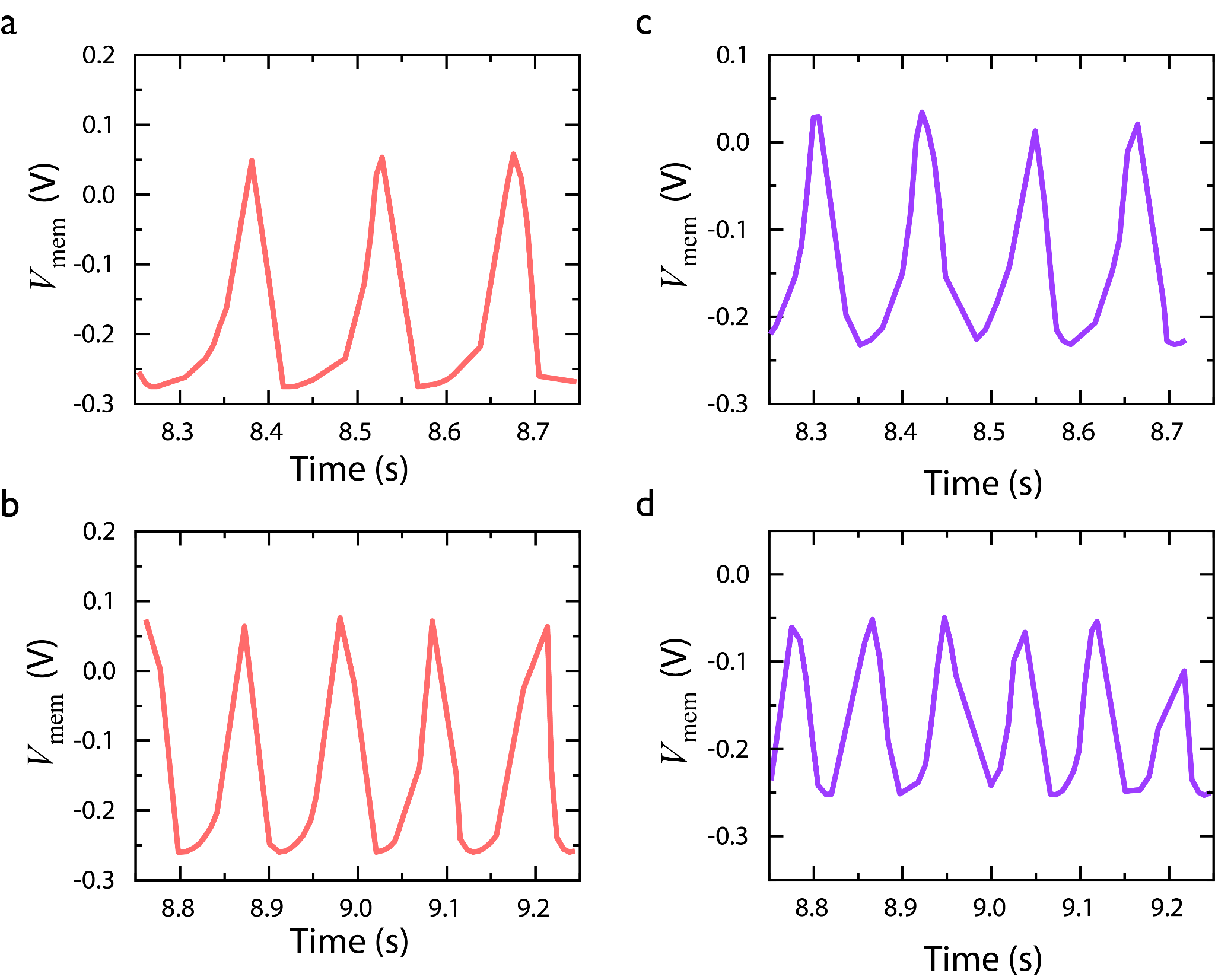


**Figure 15: Spiking traces from the spiking HH-circuits a,** vOECT BBL HH-spiking circuit output in response to 2 µA current input (*W*=25 µm). **b,** vOECT BBL HH-spiking circuit output in response to 10µA current input (*W*=25 µm) **c,** vOECT BBL/PEDOT HH-spiking circuit output in response to 2µA current input (*W*=50 µm). **d,** vOECT BBL/PEDOT HH-spiking circuit output in response to 10µA current input (*W*=50 µm).


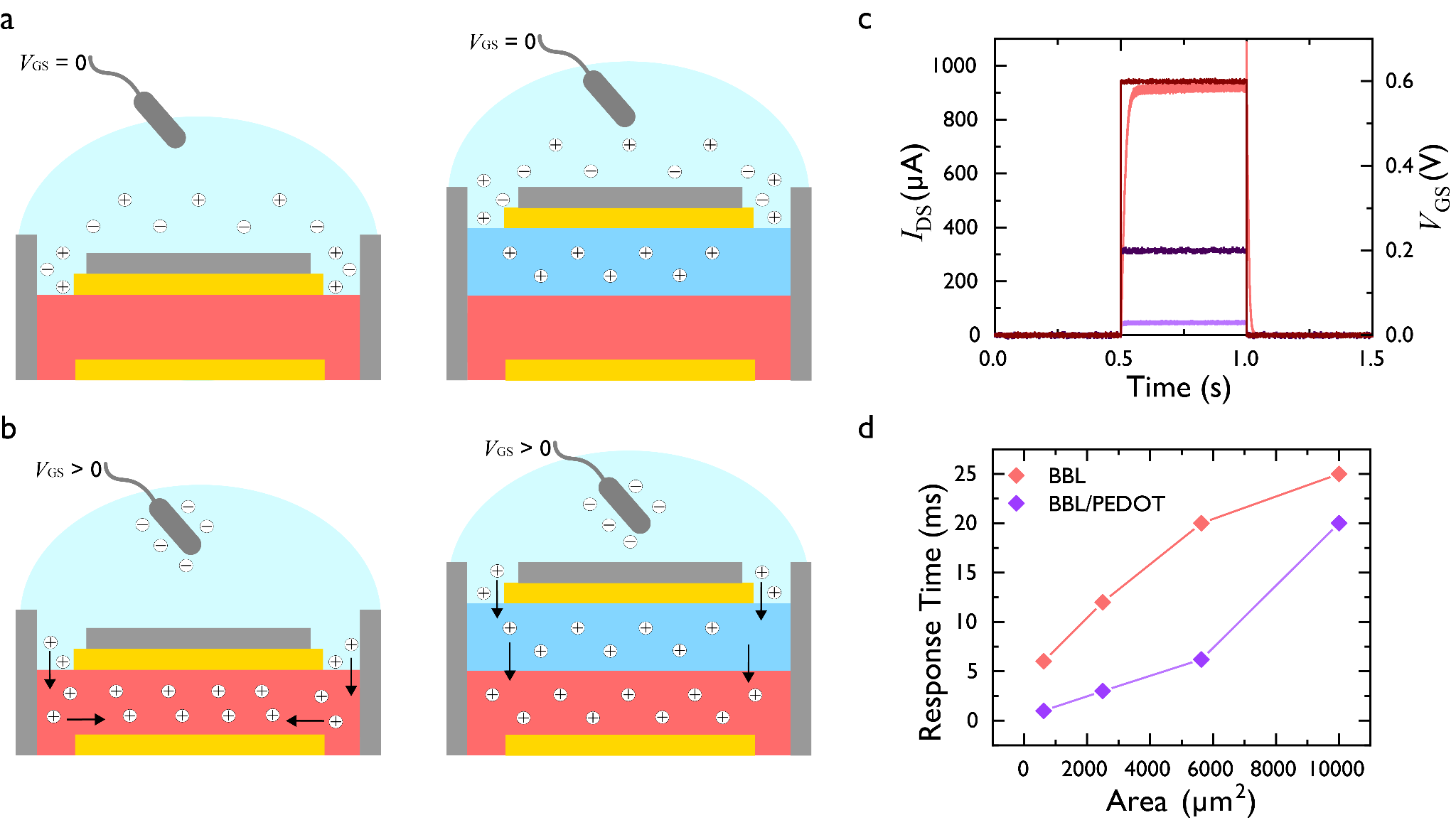


**Figure S16: Response Time comparison between the BBL and BBL/PEDOT vOECTs a,** Diagram detailing the position of ions under no gate voltage conditions. **b,** Diagram detailing the movement of ions during positive gate voltages. **c,** Example response time measurement of a BBL/PEDOT bilayer vOECT (*I*_DS_ is light purple, *V*_GS_ is dark purple) and BBL vOECT (*I*_DS_ is light red, V_GS_ is dark red) (*W*=50 µm, *V*_DS_ = 0.15 V). **d,** Scaling of the response time of BBL devices compared to BBL/PEDOT devices as a function of device area (*W*^2) (n=1).


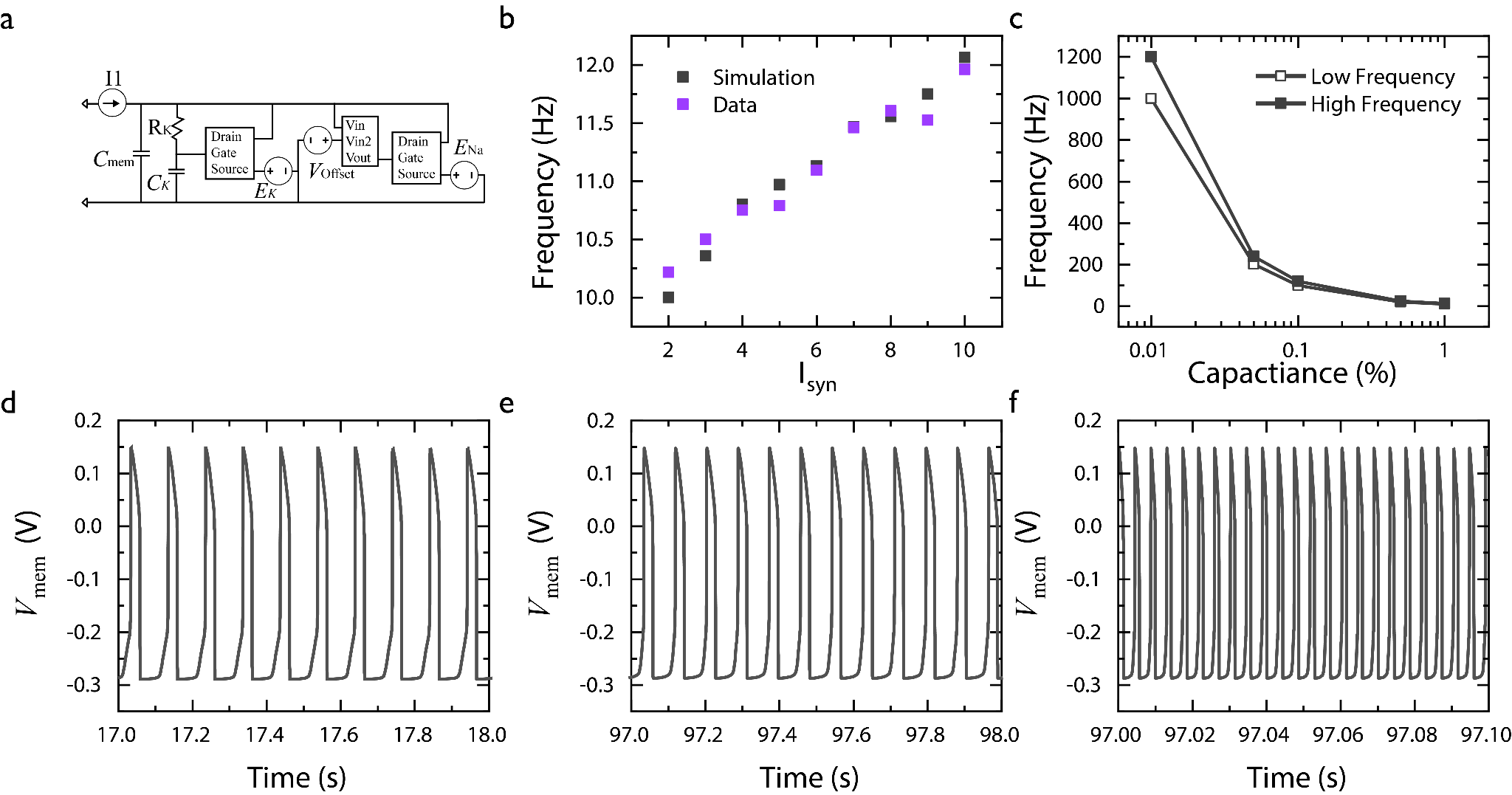


**Figure S17: Increasing the Frequency of the spiking circuit. a,** Circuit model used for simulations using the BBL-PEDOT circuit simulation model from SI Note 3. **b,** Input current scaling of frequency response for the model compared to the data. **c,** The high and low input spiking frequencies are displayed as all capacitances within the circuit are simultaneously reduced to the corresponding percentage of their initial values. **d,** Example spiking traces from the low current (2µA) input for the spiking circuit model. **e,** Example spiking traces from the high current (10µA) input for the spiking circuit model. **f,** Example spiking traces from the physiological spiking frequency (5% of the starting capacitance).


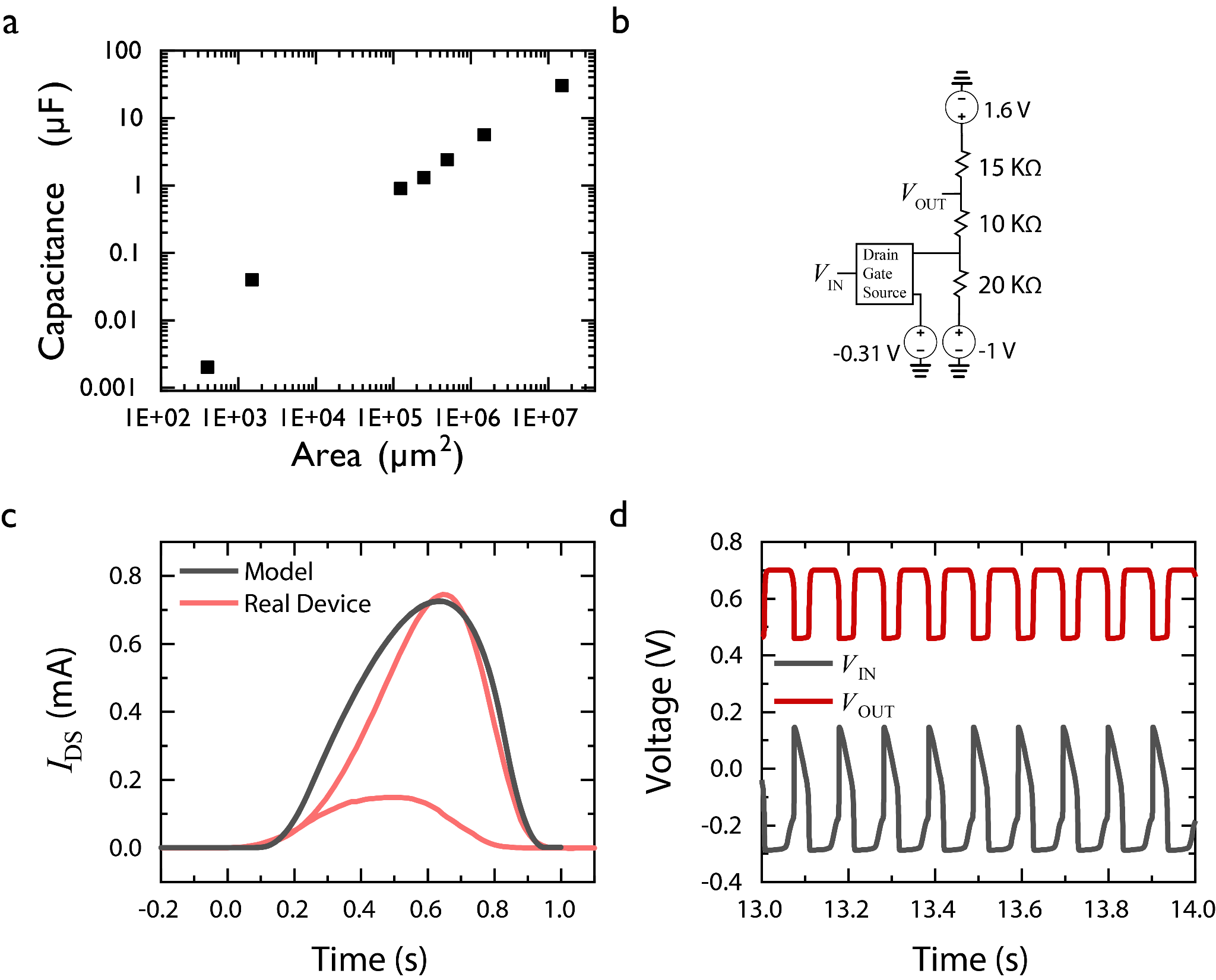


**Figure S18: Components of an all-organic spiking circuit a,** Capacitance scaling of spin-coated PEDOT:PSS capacitors with area of each plate. **b,** Circuit design for an organic inverting element. **c,** Comparison of the lateral BBL device transfer curve (*W*=2100 µm, *L*=5 µm, *V*_DS_=0.15 V) and the modeled BBL device used in the circuit model of the organic inverting element. **d,** Simulation-based spiking output and the output of the organic inverting element during an input current of 10µA.

**
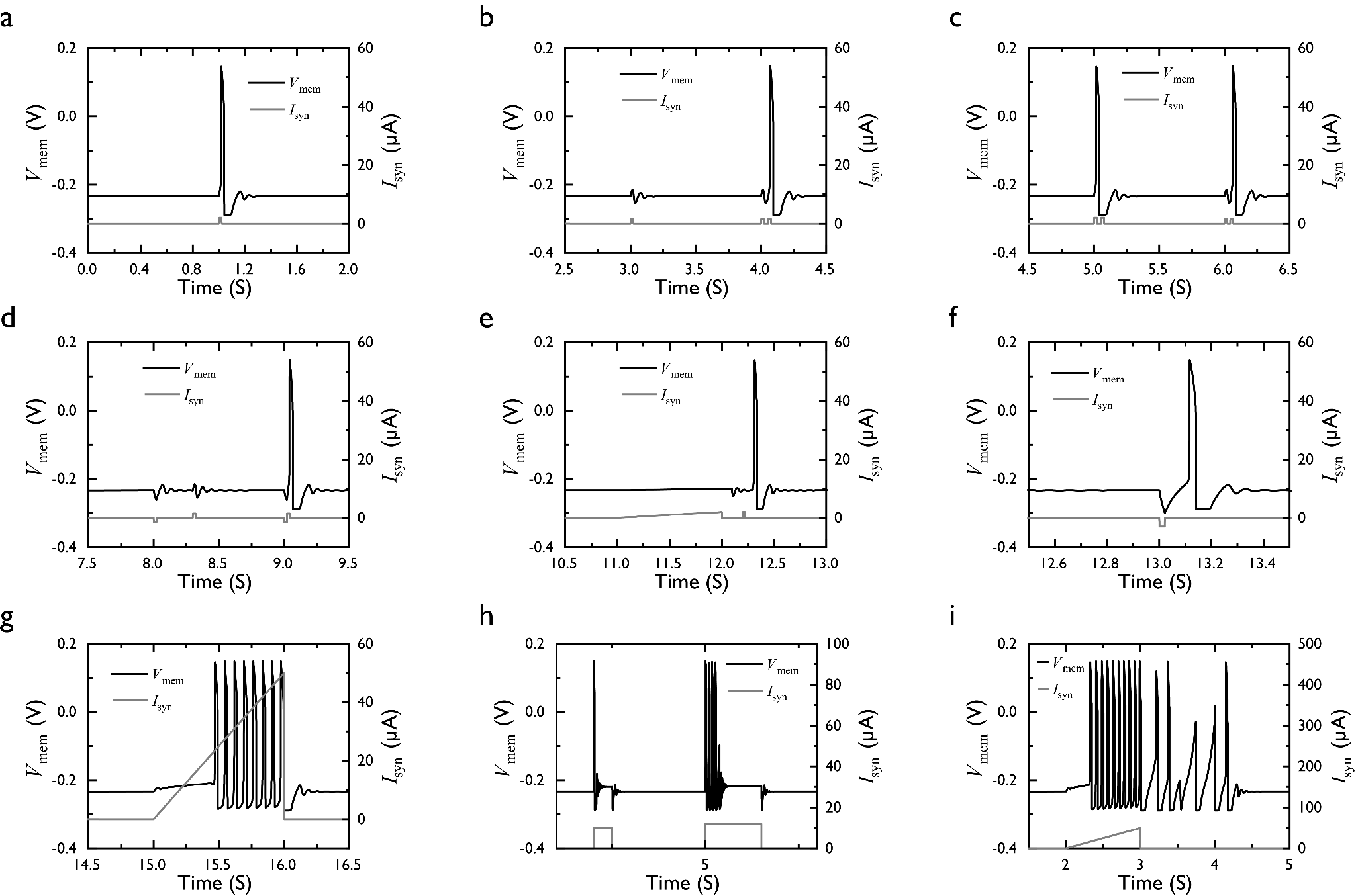
**

**Figure S19: Simulated Features of a BBL/PEDOT bilayer vOECT HH-circuit. a,** Demonstration of the spike latency within the circuit. **b,** Demonstration of the integration capabilities of the spiking circuit to sum subthreshold inputs into a spike. **c,** Demonstration of the circuit to exhibit both a refractory period (the first spike), and resonance (the second spike). **d,** Demonstration of the circuit to exhibit threshold variability depending on closely timed inputs. **e,** Demonstration of the circuit to exhibit neural accommodation, and thus a preference for sharp sudden inputs. **f,** Demonstration of the circuit to exhibit rebound spiking when a strong negative input is applied. **g,** Demonstration of Class 2 spiking in response to increased current input. **h,** Demonstration of Phasic Spiking and Phasic Bursting, as a result of the *V*_OFFSET_ increasing to 0.49 V. **i,** Demonstration of Class 1 spiking when *V*_OFFSET_ is reduced to 0.457 V

**Table S1: Metrics of the Spiking Circuit**

|  | Circuit elements | Device Footprint [µm] | Ion-  based modul-ation | Spike Amplitude [mV] | Frequency [Hz] | Power [µW]/ Energy consumption per spike [pJ] | Tunable threshold |
| --- | --- | --- | --- | --- | --- | --- | --- |
| Silicon CMOS[^11–14^](https://www.zotero.org/google-docs/?Gu0hYa) | 14 to 30+ transistors and several passive components | 0.065 | No | 120-3000 | 100 - 1000000 | 8-60 / 0.1- 1000 | Yes |
| Mott-  Memristors[^15,16^](https://www.zotero.org/google-docs/?CgGi7x) | 2 memristors  3 resistors  3 capacitors | 0.110 x 0.110 - 0.6 x 0.6 | No | 400-1200 | 10000-50000 | 2000 / 10000 | Partial |
| 2D Gaussian heterojunction[^8^](https://www.zotero.org/google-docs/?blifHD) | 3+ transistors  2+ resistors  1-3 capacitors | 100 x 100 | No | 400 | <1 | 1.25 / 250000 | Yes |
| OFET (LIF)[^17^](https://www.zotero.org/google-docs/?nJoot4) | 5 transistors  2 capacitors | 1000 x 2000 | No | 3000 | 1-5 | 40 / 20000000 | Yes |
| Complementary OECT (LIF)[^9^](https://www.zotero.org/google-docs/?AwCMYI) | 5 transistors  2 capacitors | 2000 x 200 | Yes | 500 | 0.25 | 15 / 15000000 | Yes |
| Previous lateral OECT (HH)[^2^](https://www.zotero.org/google-docs/?Asma1M) | 3 Transistors  2 Resistors  1 Capacitor | 400 x 10 | Yes | 100-200 | 100 | 58.6 / 175000 | Partial |
| **Bilayer vOECT (This work)** | **3 Transistors**  **1 Resistor**  **2 Capacitors** | **50 x 50** | **Yes** | **100-300** | **0.5-25** | **8.2 /1090000** | **Yes** |
| Biological Neuron[^18,19^](https://www.zotero.org/google-docs/?O3MWVO) | N/A | ~50 (soma) | Yes | ~120 | 0.5 - 500 | 100 | Yes |

**SI Note 3:**

To implement the Bilayer vOECT devices into an Hodgkin-Huxley spiking circuit, a breadboard was used to enable node connections between commercially available capacitors, resistors, and operational amplifiers (TL084CN) to the vOECT devices, which were on separate glass slides each containing electrolyte and a ag/agcl gate electrode pellet. The circuit was then powered using two keithley as described in the methods section.

BBL/PEDOT bilayer vOECT was investigated for its applications in Hodgkin-Huxley Spiking circuits. Details regarding the devices used for the spiking circuit and the spikes metrics produced are presented in **Figure S14** and **Figure S15**. It is of interest to note that in **Figure S15** the lowest *I*_syn_ generating spikes is 2 µA, whereas in **Figure 4** the lowest is 3 µA. At the capacitance value used for this circuit, the faster charging of the capacitor enabled lower currents to generate spiking, which was not the case for all capacitors, and as such 3 µA reliably produced spiking at every capacitance for the direct comparison seen in **Figure 4**. Additionally, the 50x50 µm BBL/PEDOT Bilayer device was used instead of the 25x25 µm BBL/PEDOT device due to the insufficient ON/OFF ratio of the 25x25 µm device to enable reliable spiking. Furthermore, the 25x25um devices were the smallest device size created due to difficulties during peel-off in smaller devices, that lead to incomplete channel layers and thus electrical shorts between the top and bottom electrodes. Therefore, the 25x25 µm BBL devices were utilized in the vOECT spiking circuit due to the small footprint and increased speed compared to the other BBL vOECT dimensions (**Figure S16**).

As previously discussed in Harikesh et al., the intrinsic properties of the anti-ambipolar OECT enable replication of neuron spiking activity by mimicking the key activation and inactivation parameters of the Na and K channels presented in the classical Hodgkin-Huxley equations[^2,20,21^](https://www.zotero.org/google-docs/?qLCCJ1). The bilayer anti-ambipolar OECTs can replicate the relationship between these gating variables and the conductance of the channel due to the OFF-ON-OFF behavior of the device’s transfer curve, and the OFF-ON behavior of the Gate-Drain connected voltage sweep (**Figure 4**).Specifically, the K channel behavior can be replicated with the traditional linear regime OECT equations where the activation gating variable is akin to the current dependance on applied and threshold voltage[^2,20,21^](https://www.zotero.org/google-docs/?WWOCqC). On the other hand, the Na channel behavior can be replicated as an accumulation mode and depletion mode material if *V*_GS_ is before or after the peak, respectively. This then replicates the activation and inactivation gating variable based on the changes in mobility and threshold voltage when switching from OFF to ON and ON to OFF, respectively[^2,20,21^](https://www.zotero.org/google-docs/?SS29ei). For a detailed explanation of the relationship between device equations and the Hodgkin-Huxley equations, we refer the reader to Supplementary Notes 3 and 4 within Harikesh et al[^2,20,21^](https://www.zotero.org/google-docs/?AgaXTF).

Although additional circuit components can be added to enable more refined biological functions, we limited the application of these devices to the traditional HH circuit model, as only the Na channel necessitates anti-ambipolar transfer characteristics [^2,7,20,22^](https://www.zotero.org/google-docs/?sXUJBx). Interestingly, the BBL/PEDOT bilayer vOECT spiking circuit showed increased frequencies compared to the BBL vOECT spiking circuit, and thus the response time and capacitance of the single devices composing the circuits were investigated **(Figure S16)**. The BBL/PEDOT bilayer vOECTs show a decreased response time in comparison to the BBL vOECTs, as a result of the top PEDOT layer acting as an ion reservoir by facilitating the ion movement. Moreover, the top PEDOT layer also helps to control the primary ion transport direction allowing a vertical flow with decreased Ion travel distances, compared to lateral flow **(Figure S16)**[^23^](https://www.zotero.org/google-docs/?OGiX1S).

Based on the results of this spiking circuit and the employed devices, an LTSpice simulated circuit was developed using the same device circuit design discussed in SI Note 2 but optimized to reflect the measured peak currents and capacitance of a *W*=50 µm BBL/PEDOT device **(Figure S4, S17)**. Using this simulation, the impact of capacitance on spiking frequency was explored by reducing the capacitance of every component simultaneously to a percentage of the original value as seen in **Figure S17 c**. By reducing the capacitances in half order of magnitude steps (ie 50, 10, 5, 1) of the starting values (*C*_mem_=*C_k_*=0.47uF, *C*_OECT_ = 0.135uF, 0.010uF), the HH-spiking circuit is capable of producing spiking widths within the physiological range of 3-5ms at 5% of the initial capacitance values, promising future central nervous system interfacing **(Figure S17)**[^18,20,24^](https://www.zotero.org/google-docs/?yzlGg8). Additional reductions in capacitance can lead to higher frequencies, and thus decreased power consumption, but reduced bio-interfacing opportunities for applications in novel computing hardware[^2,7,20,22^](https://www.zotero.org/google-docs/?ox15ns).

Additionally, the path towards an all-organic spiking circuit was explored using the simulated spiking circuit. Primarily, PEDOT:PSS (with 6% EG and 1%Gops) capacitors were fabricated to determine the scaling of capacitance with area of these devices **(Figure S18)**. This ensured that organic capacitors within the range of capacitances needed could be fabricated. For the resistors, there exists a range of options including carbon-based inks or patterned and insulated PEDOT:PSS or other organic semiconducting material channels[^25–27^](https://www.zotero.org/google-docs/?2kOsV4). Finally, the inverting operational amplifier can be replicated using a lateral BBL OECT as seen in **Figure S18**. For this circuit, a lateral BBL OECT was simulated using the same equivalent circuit as the BBL/PEDOT bilayer vOECT, except capacitance, turn off, and current was adjusted to match the real device data **(Figure S18c)**. Using this simulation, the circuit depicted in **Figure S18b** was used to replace the simulated spiking circuit’s inverting amplifier in **Figure S17**, leading to the spiking output seen in **Figure S18d**. Therefore, it is possible with additional optimization and fabrication to create an all-organic spiking circuit for large scale integration and implementation, but the need to avoid cross-talk in between each organic component (separate gating mechanisms) remains a technical challenge that significantly hinders size reduction of the circuit[^28^](https://www.zotero.org/google-docs/?0jR3el). For reference, a comparison between the spiking circuit demonstrated in this work and previous spiking circuits can be seen in **Table S1**.

Finally, the range of known OECT-based HH neuron characteristics is simulated using the circuit in **Figure S17**, thereby confirming the perseverance of complex firing behaviors in the bilayer vOECT neurons despite primarily using tonic firing **(Figure S19)**. Further advancements in the complexity of spiking behaviors in OECT-based HH neurons could be achieved via inclusion of additional input circuitry as demonstrated in Yi et al. as well as improvements in p-type material selection, which can be optimized to increase threshold stochasticity and include adaptive threshold capabilities, such as those demonstrated in PEDOT:PTHF[^15,29^](https://www.zotero.org/google-docs/?wBNOAw).


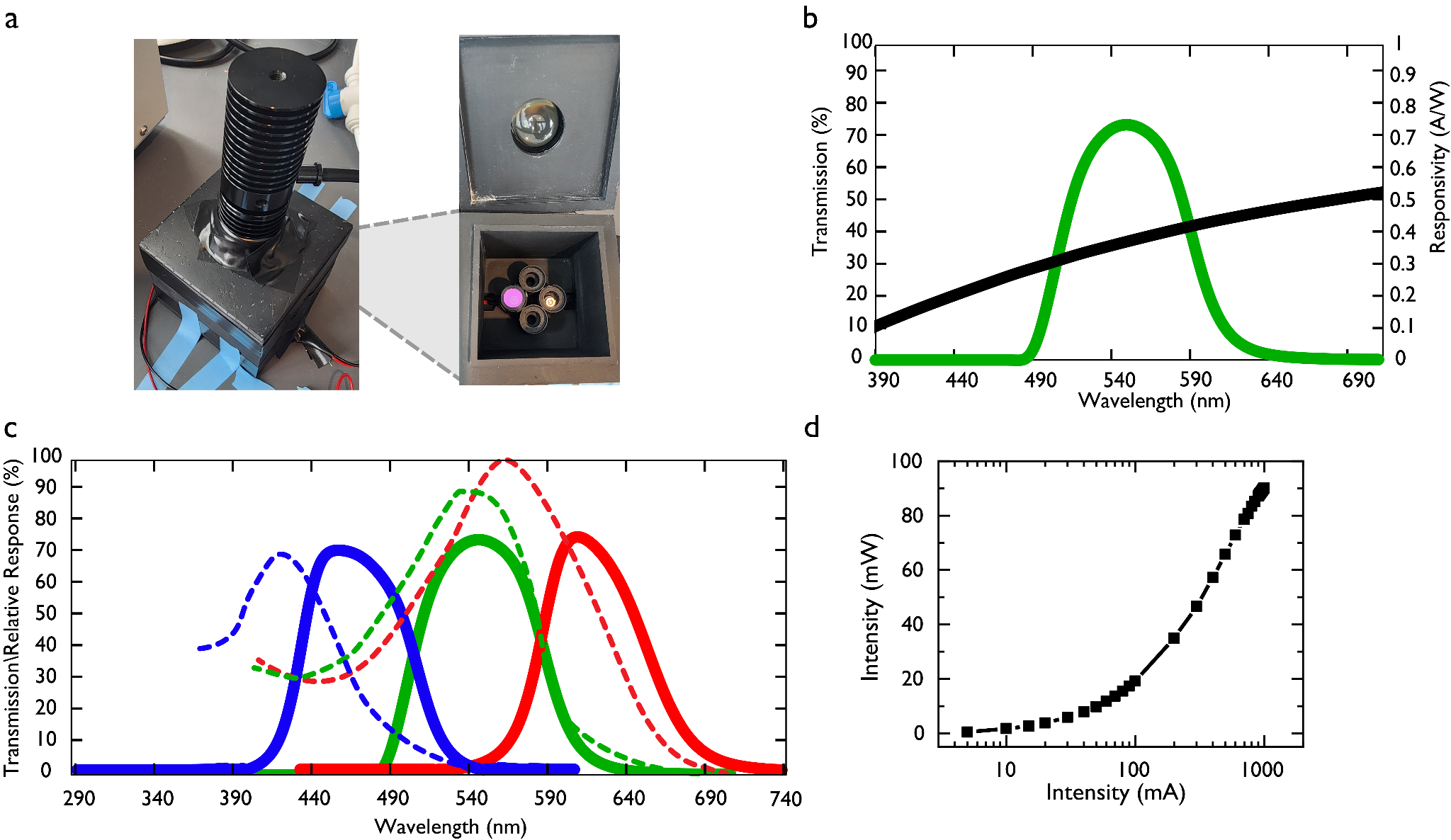


**Figure S20: Phototransduction and Wavelength-specificity a,** 3D-printed setup for testing optical filters and photodiodes. **b,** Transmission of green optical (550nm CWL, 80nm FWHM Edmund optics filter #65-735) filter and responsivity of photodiode(FD11A - Si Photodiode Thorlabs). **c,** Transmission of red (600nm CWL, 80nm FWHM, Edmund optics filter #65-736), blue (450nm CWL, 80nm FWHM, Edmund optics filter #65-733), and green optical filters (solid lines of corresponding color) compared to biological relative response cones (dotted line). **d.** Measured intensity corresponding to the current supplied to the LED light source.


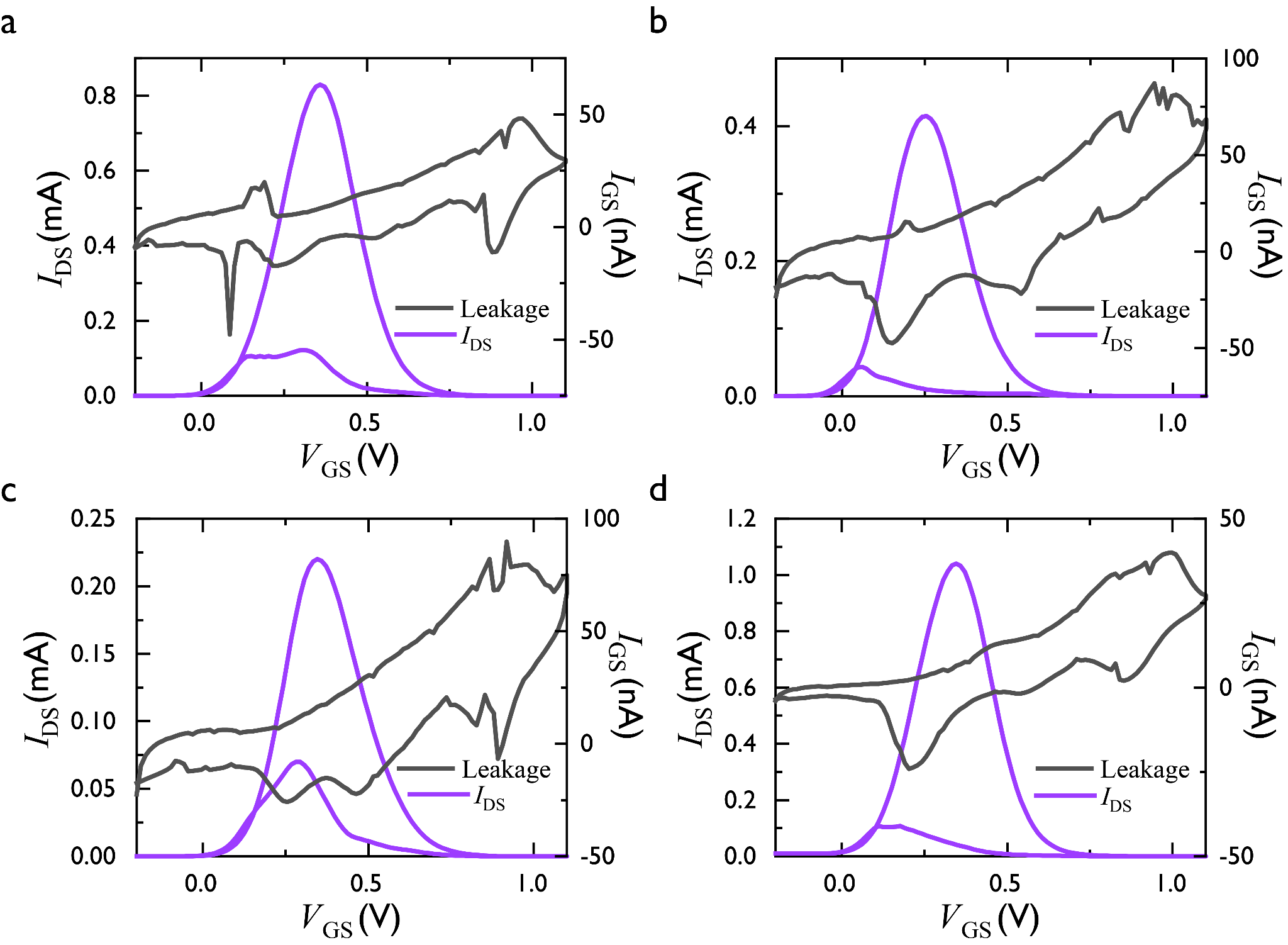


**Figure S21: BBL/PEDOT vOECT devices used in the retinal pathway a,** BBL/PEDOT device used for the rod (*W*=50 µm, *V*_DS_ = 0.15 V) **b,** BBL/PEDOT device used for the cone (*W*=50 µm, *V*_DS_ = 0.15 V). **c,** BBL/PEDOT device used for the K-channel (*W*=50 µm, *V*_DS_ = 0.15 V). **d,** BBL/PEDOT device used for the Na Channel (*W*=50 µm, *V*_DS_ = 0.15 V)


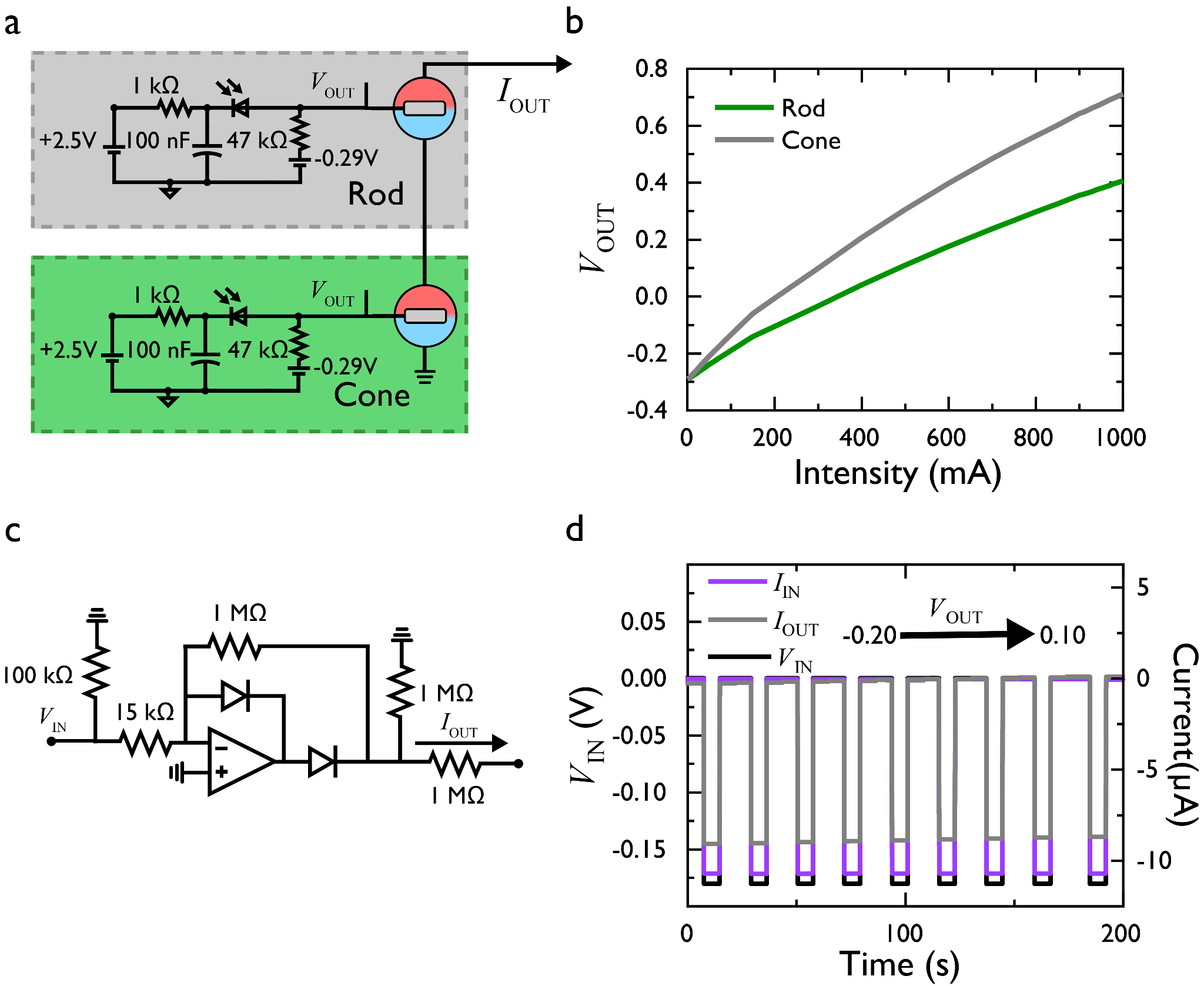


**Figure S22: Supporting circuitry for the photosensitive cells and bipolar cells. a,** Circuit for retinal cone/rod transduction. **b,** Voltage at the output for rod (gray) or cone (green) transduction circuit under all light conditions. **c,** Circuit for the precision rectifier (ideal diode) used between the horizontal cell output and the input of the spiking circuit. **d,** Characteristics of the rectifier circuit as *V*_IN_ switches between 0 and -0.18V and Vout sweeps over the spike amplitude.


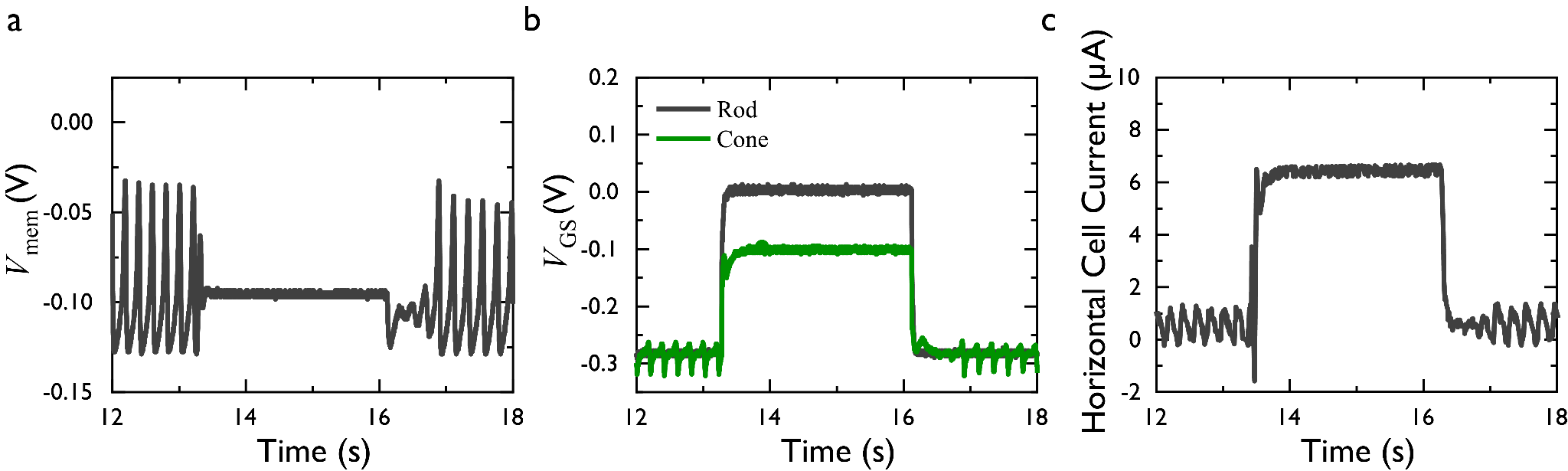


**Figure S23: Results without the bipolar cell. a,** Spiking data from a retinal pathway circuit set to spike only continuously as light is applied. **b,** Voltage at the gates of the rod (gray) and cone (green) devices during light application. **c,** Output of the Horizontal Cell (AND gate) during light application.


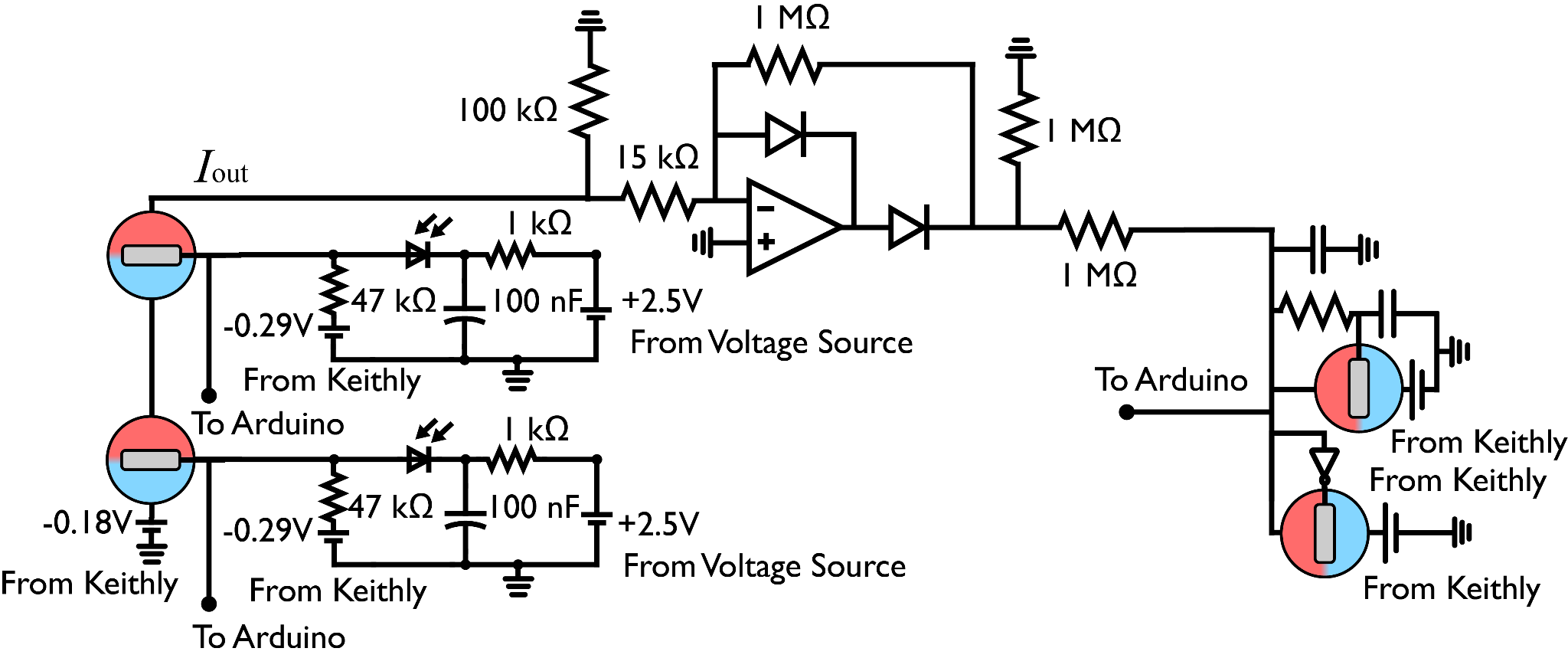


**Figure S24: Complete Circuit with Recording Nodes** Diagram detailing the circuit used during the retinal pathway replication, with appropriate source meter unit, arduino, and voltage source channel locations.


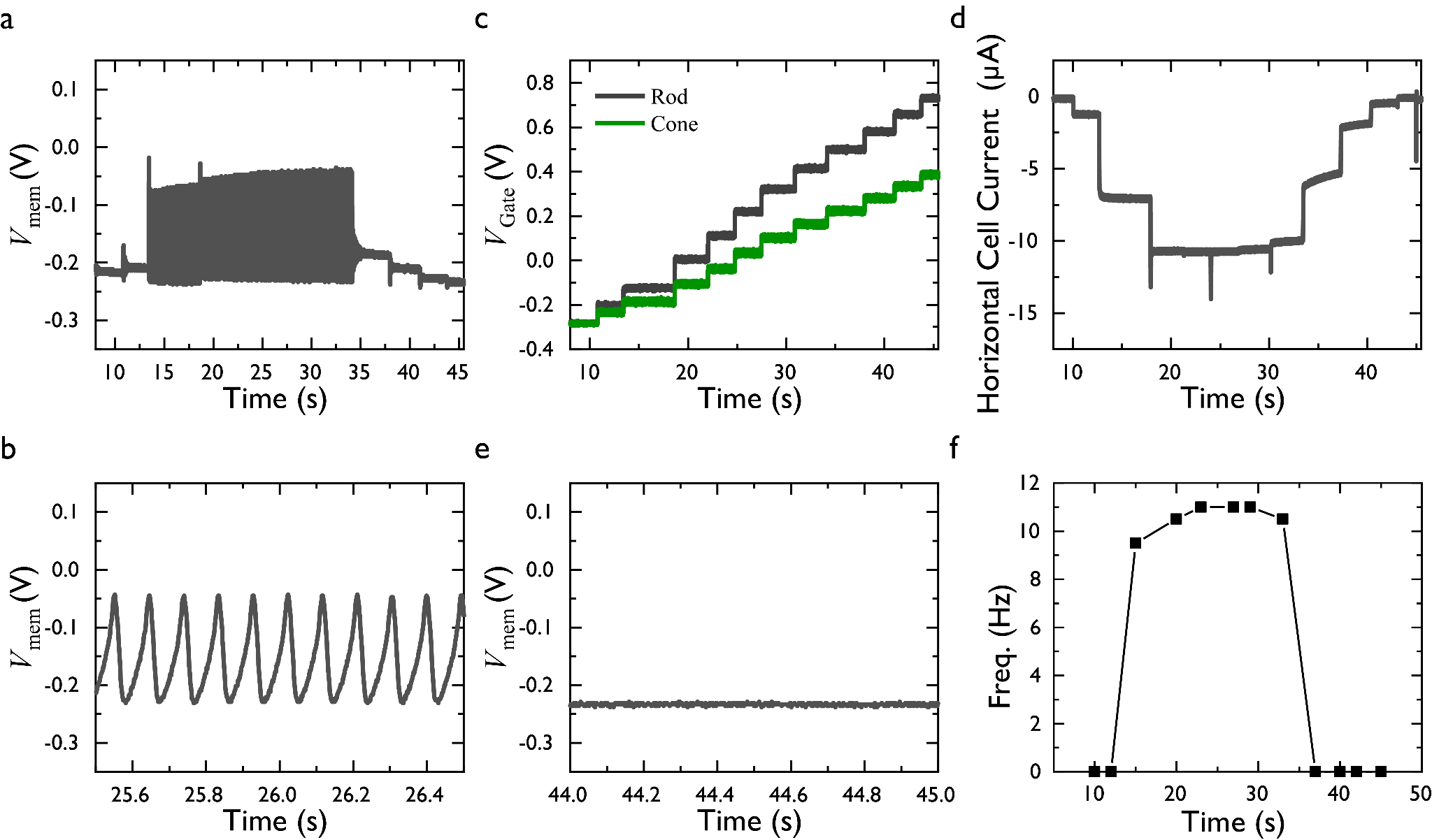


**Figure S25: Dynamic Light Output Without Starting Spikes a,** Spiking data from a retinal pathway circuit set to spike only during light exposure under all light conditions as light gradually increases. **b,** Spike traces from the highest frequency condition. **c,** Voltage at the gates of the rod (gray) and cone (green) devices over all light conditions as seen in **Figure 5**. **d,** Output of the Horizontal Cell (AND gate) over all light conditions. **e,** Spike traces from the lowest frequency (highest light) condition. **f,** Frequency of the spiking over all light conditions.


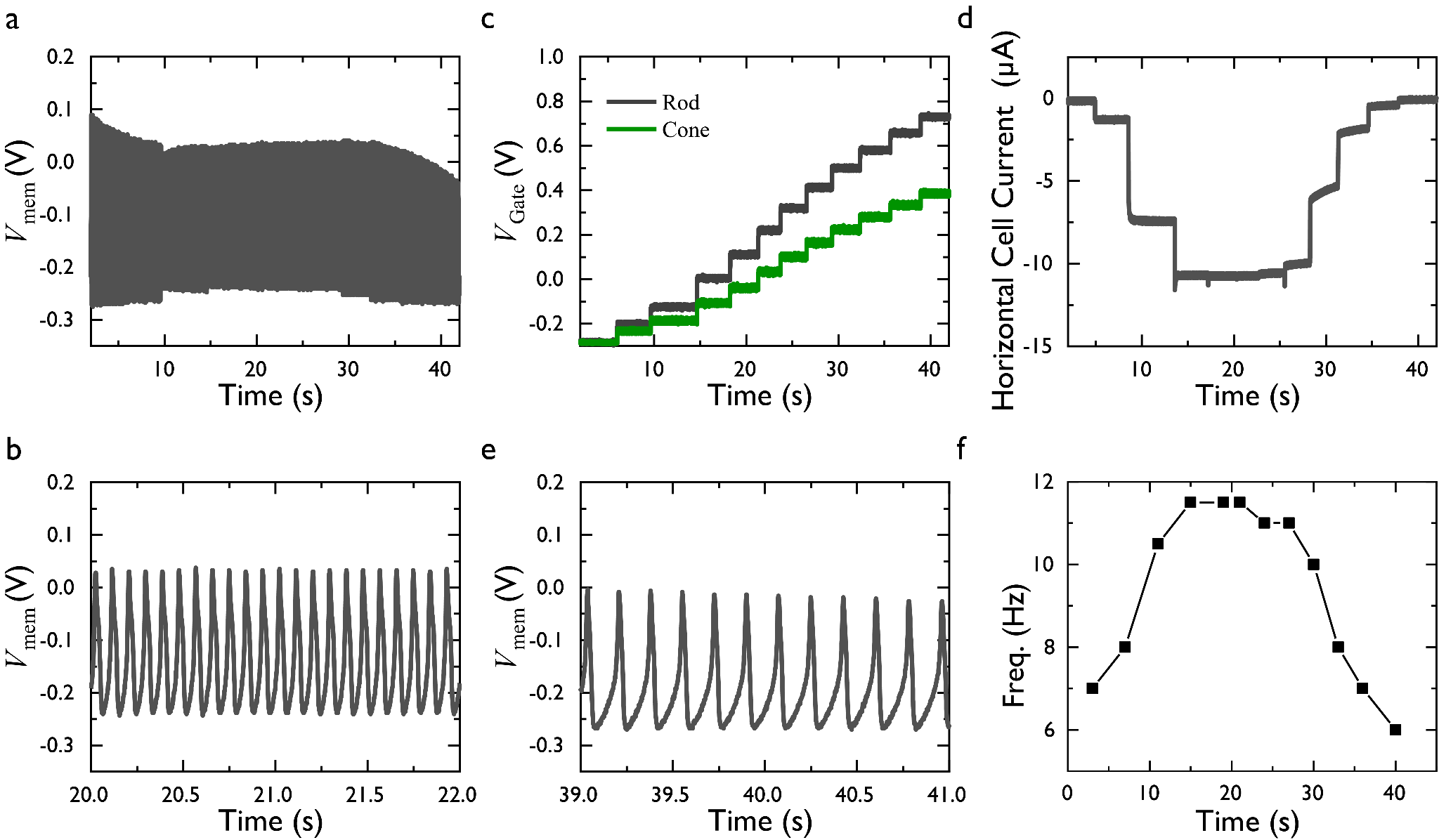


**Figure S26: Dynamic Light Output With Starting Spikes a,** Spiking data from a retinal pathway circuit set to continuously spike under all light conditions as light gradually increases. **b,** Spike traces from the highest frequency condition. **c,** Voltage at the gates of the rod (gray) and cone (green) devices over all light conditions as seen in **Figure 5**. **d,** Output of the Horizontal Cell (AND gate) over all light conditions. **e,** Spike traces from the lowest frequency (highest light) condition. **f,** Frequency of the spiking over all light conditions.


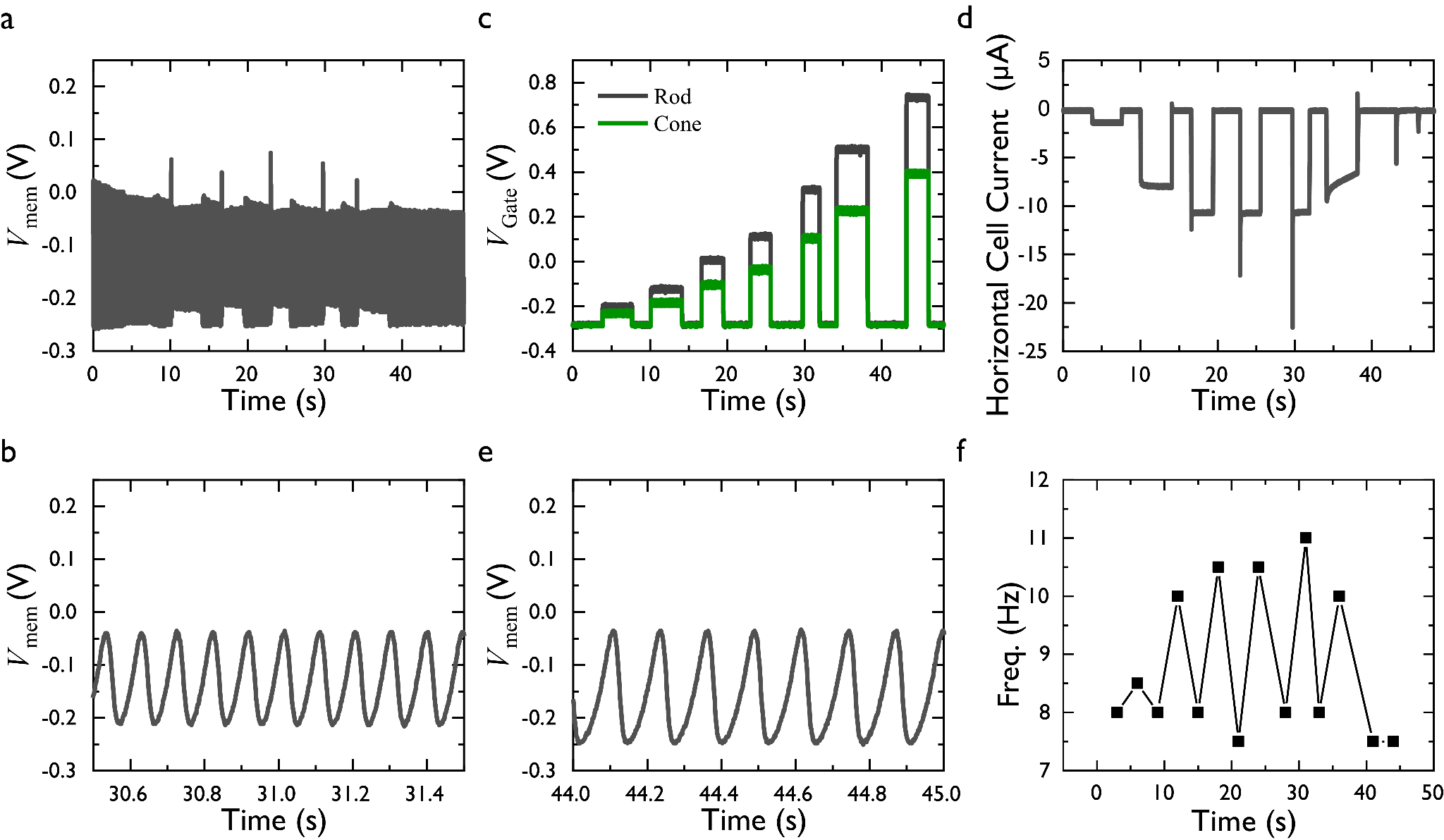


**Figure S27: Static Light Output With Starting Spikes a,** Spiking data from a retinal pathway circuit set to continuously spike under all light conditions as light switches from specific intensities and the no-light state. **b,** Spike traces from the highest frequency condition. **c,** Voltage at the gates of the rod (gray) and cone (green) devices over all light conditions as seen in **Figure 5**. **d,** Output of the Horizontal Cell (AND gate) over all light conditions. **e,** Spike traces from the lowest frequency (highest light) condition. **f,** Frequency of the spiking over all light conditions


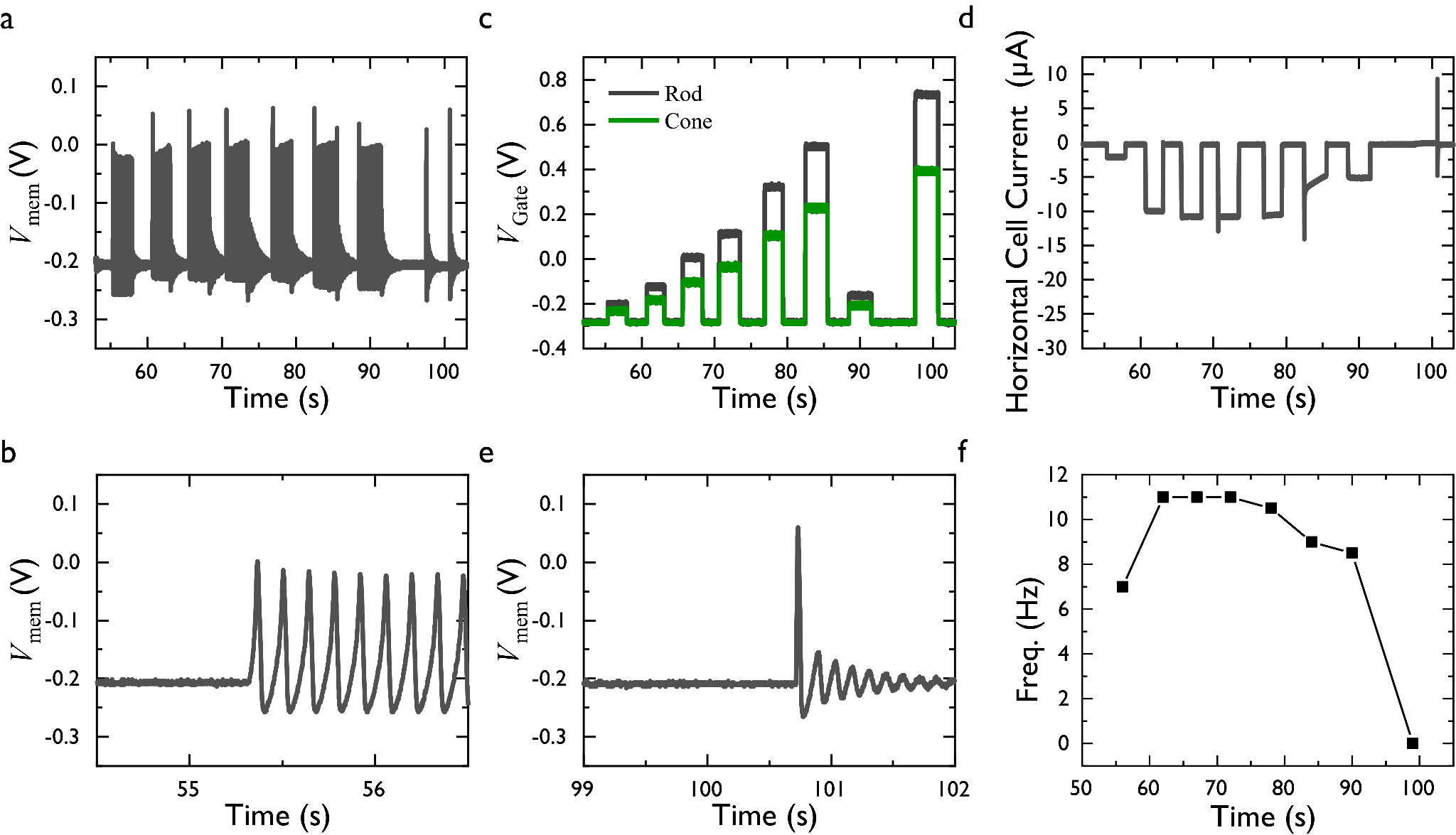


**Figure S28: Static Light Output Without Starting Spikes a,** Spiking data from a retinal pathway circuit set to spike only during light exposure under all light conditions as light switches from specific intensities and the no-light state. **b,** Spike traces from the highest frequency condition. **c,** Voltage at the gates of the rod (gray) and cone (green) devices over all light conditions as seen in **Figure 5**. **d,** Output of the Horizontal Cell (AND gate) over all light conditions. **e,** Spike traces from the lowest frequency (highest light) condition. **f,** Frequency of the spiking over all light conditions


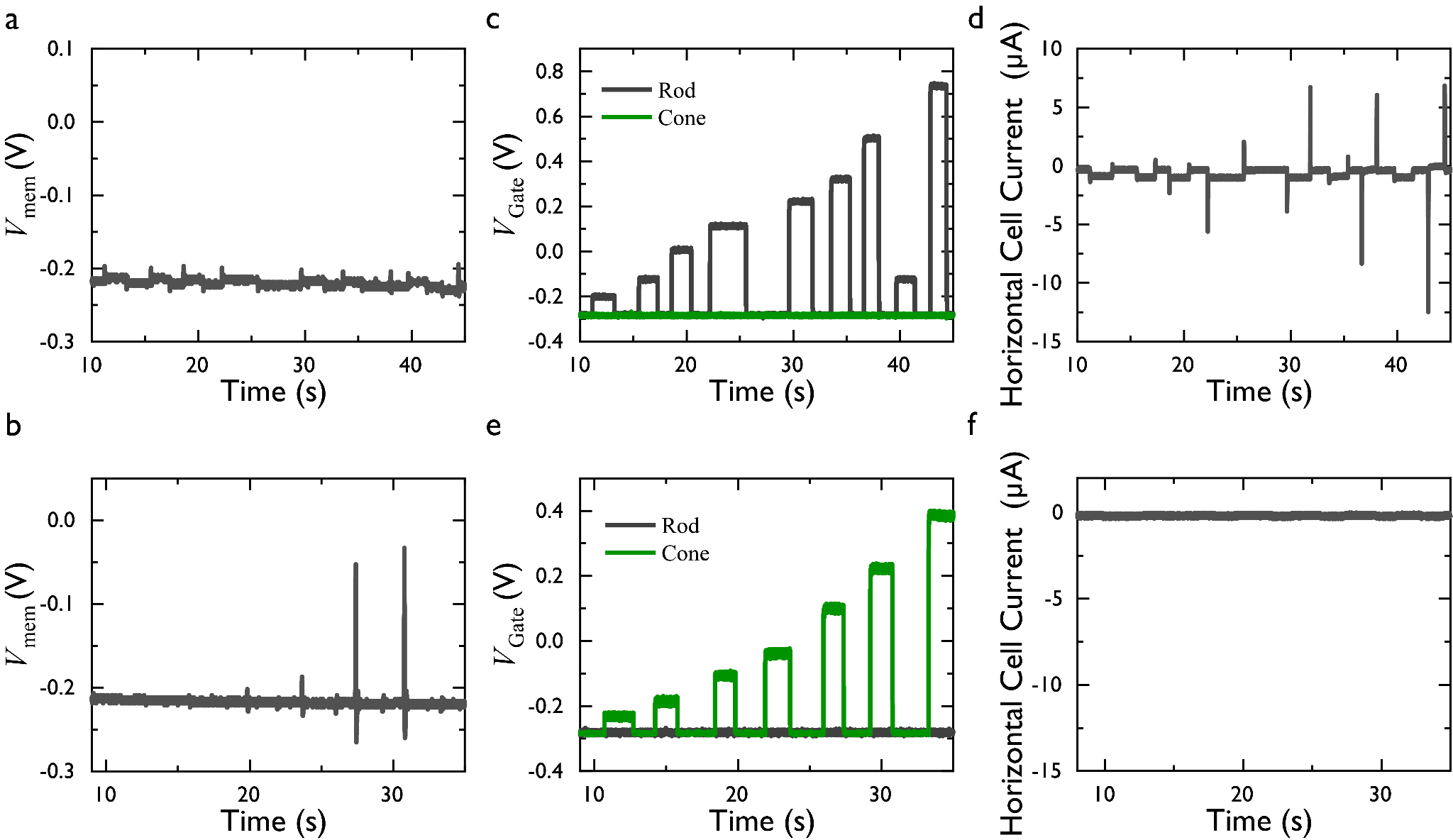


**Figure S29: Removal of Rods or Cones from Horizontal Cell a,** Spiking data from a retinal pathway circuit set to spike under all light conditions as light switches between specific intensities and no-light states, but the photodiode replicating the cone transduction is disconnected. **b,** Spiking data from a retinal pathway circuit set to spike under all light conditions as light switches between specific intensities and no-light states, but the photodiode replicating the rod transduction is disconnected. **c,** Voltage at the gates of the rod (gray) and cone (green) devices over all light conditions as seen in **Figure 5**, with the cone photodiode disconnected. **d,** Output of the Horizontal Cell (AND gate) with the cone photodiode disconnected over all light conditions. **e,** Voltage at the gates of the rod (gray) and cone (green) devices over all light conditions as seen in **Figure 5**, with the rod photodiode disconnected. **f,** Output of the Horizontal Cell (AND gate) with rod photodiode disconnected over all light conditions.


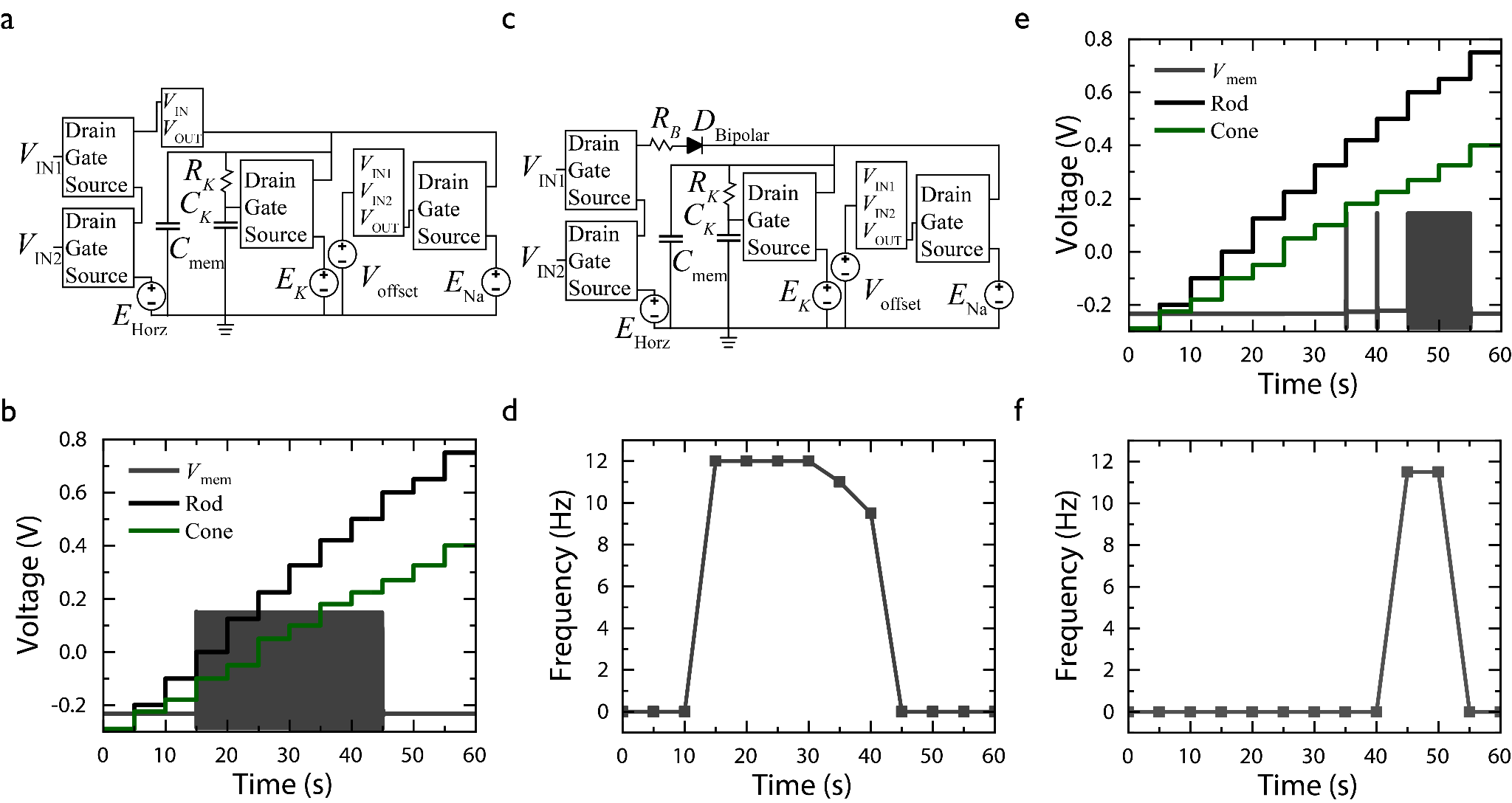


**Figure S30: Simulation of the Retinal Pathway with reduced components a,** LTSpice circuit used to model the retinal pathway. **b,** Spiking output of the horizontal cell model over all light conditions. **c,** LTSpice circuit used to model the retinal pathway with a schottky diode for the bipolar cell, *R_B_* set to 10 Kohms, and *E*_Horz_ set to 0.2 V. **d,** Frequency over all light conditions for the original model. **e,** Output of the model in panel c over all light conditions. **f**, Frequency over all light conditions for the schottky diode model.


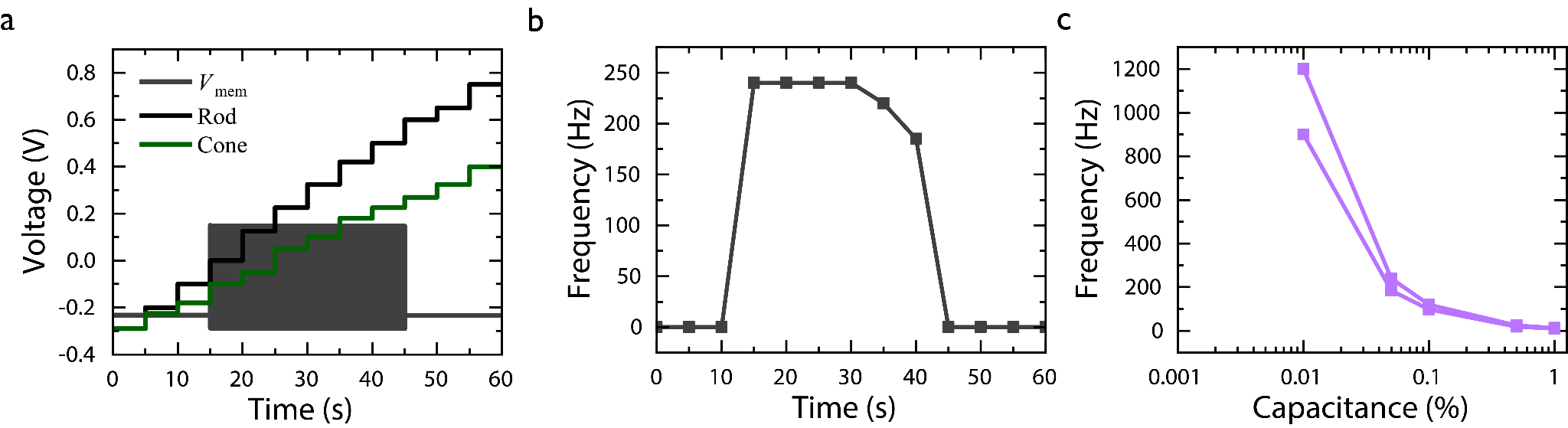


**Figure S31: Increasing the frequency of the retinal pathway a,** Output of the model in **Figure S30a** over all light conditions, when all capacitances are reduced to 5% of its initial capacitance. **b,** Frequency over all light conditions when all capacitances are reduced to 5% of its initial capacitance. **c,** Scaling of frequency range for the retinal pathway as all capacitances are reduced by the corresponding percentage.


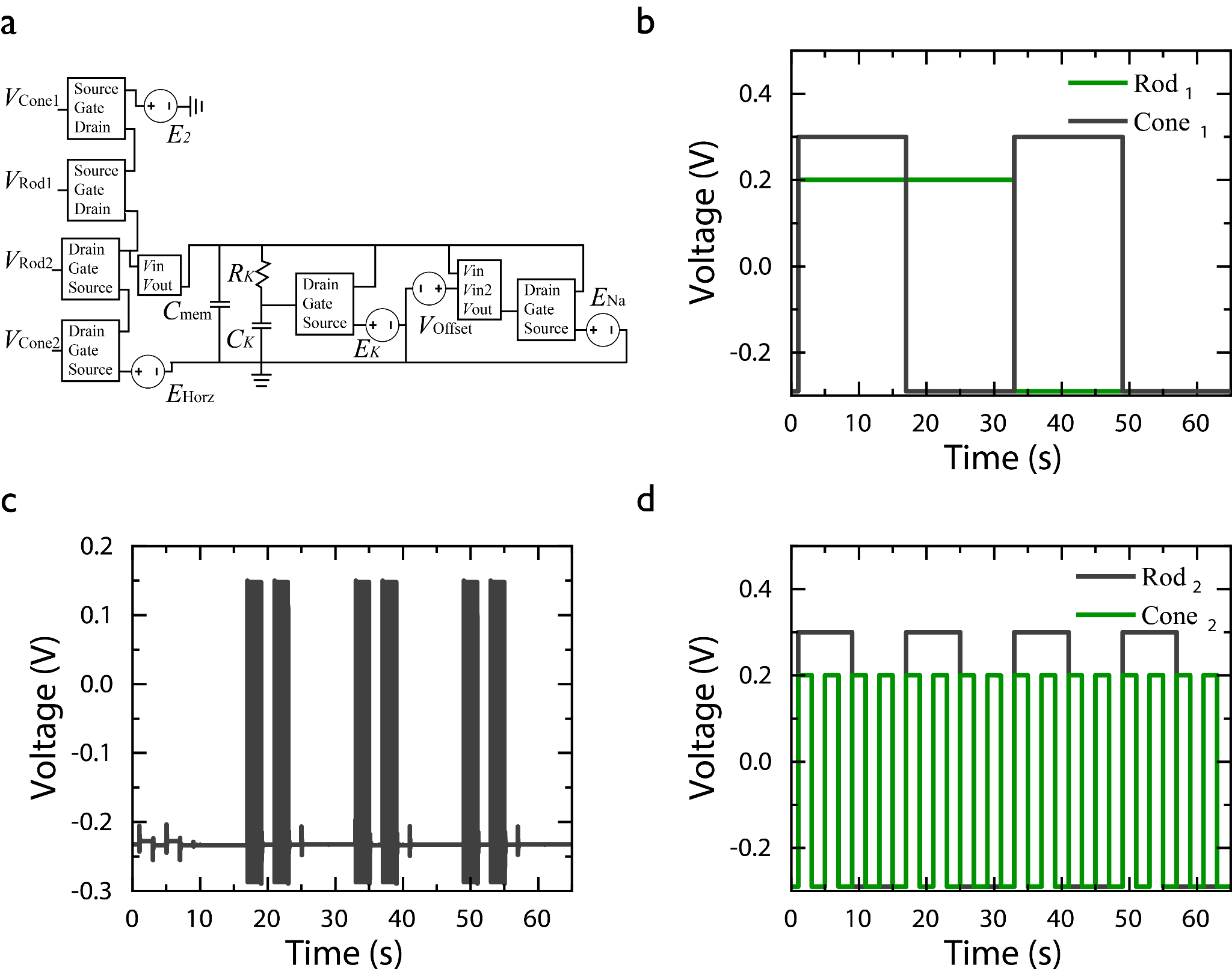


**Figure S32: Multiple Horizontal Cells influencing the activity of a single bipolar cell and spiking neuron. a,** LTSpice circuit used to model multiple horizontal cells converging at the same bipolar cell, where *E_2_* is set to 0.2V and *E*_Horz_ is -0.18V. **b,** Voltage of the photoreceptors connected to the first horizontal cell. **c,** Spiking output of the retinal ganglion cells as light is applied to each of the photoreceptors as seen in panels b and d. **d,** Voltage of the photoreceptors connected to the second horizontal cell.

**SI Note 4:**

With reference to the retinal pathway presented in **Figure 5, Figure S20** illustrates the system used for isolating and stimulating the photodetectors. The photodetectors (see Methods) show a mostly constant responsivity within the visible light region and with the use of optical filters showing bandpass behavior, artificial cones and rods with wavelength sensitivity have been replicated **(Figure S20)**. Alternatively, organic photodetectors can be explored to create all-organic retinal pathways but given the limited options for physiologically-relevant wavelength sensitivity inherent to organic photodetectors, this remains as a challenge for future organic photodetectors on the path towards the development of all-organic retinal pathways[^30–34^](https://www.zotero.org/google-docs/?S0BwrH).

Using this setup for light transduction, the photodiodes were combined with the supporting circuitry in **Figure S22** for converting the detected light into an output voltage to be used as an input to the gates of the BBL/PEDOT bilayer vOECTs (horizontal cell). In addition, the characteristics of the precision rectifier used for the bipolar cell can be seen in **Figure S22** which enable unidirectional current flow into the spiking circuit for “stimulating” the neuron. Primarily it is important to note that without the bipolar cell diode, the activation of the logic gates clamps the *V*_mem_ voltage to the horizontal cell offset voltage, which prevents spiking activity **(Figure S23)**. Furthermore, the BBL/PEDOT vOECT devices used for the retinal pathway can be seen in **Figure S21**. The complete retinal pathway circuit can be seen in **Figure S24** along with the recording/stimulating nodes as a reference.

Expanding upon the data shown in **Figure 5**, **Figure S25** shows the voltages at the rod and cone, the current through the horizontal cell, and the high and low spike traces as a reference. Alternatively, the spiking circuit will spike at a constant rate by reducing the offset voltage applied to the inverting amplifier. This constant spiking rate can then be modulated with light application as seen in **Figure S26**. These results were then repeated in static switching of light conditions to demonstrate consistent results in the event of sudden light changes such as going from inside to outside, blinking, and switching on a light **(Figure S27,S28)**. Furthermore, the cone and rod photodiodes were disconnected independently under illumination, showing no spiking output and thus confirming the dependance of this retinal pathway output on wavelength specificity **(Figure S29)**. In all cases, the system was able to encode light intensity into firing intensity within the ON window of the BBL/PEDOT bilayer devices. To closely replicate the retinal bandpass and intensity encoding, a bilayer vOECT could be constructed exhibiting an asymmetric triangular transfer curve, where a linear increase in drain current with gate voltage can provide intensity encoding across a larger dynamic range, while a sharp (nearly instantaneous) switch towards the second OFF state can preserve the bandpass capabilities of the bilayer device. As such, additional material optimization would be needed to combine a p-type with high current and transconductance near threshold with an n-type exhibiting a near linear trend in drain current with gate voltage.

To explore the reduction of components and increasing frequency on retinal pathway functionality, an LTSpice model of the retinal pathway was created using the aforementioned BBL/PEDOT bilayer vOECT device models **(Figure S30)**. When devices in the model were matched to the capacitance and peak current values of the *W*=50 µm BBL/PEDOT bilayer vOECTs, the retinal pathway demonstrated behavior incredibly similar to the collected results **(Figure S30)**. Based on this model, a Schottky diode with a *V*_forward_ of 0.3V and resistor replaced the precision rectifier and showed spiking behavior over a reduced range of light **(Figure S30)**. Additionally, the offset voltage for the horizontal cell was increased to 0.2V, which created an increased *V*_DS_ and would lead to a slight decrease in stability of the BBL/PEDOT bilayer vOECT **(Figure S30)**. However, organic diodes (including Schottky diodes) can be used to create an all organic retinal pathway and enable higher density integration than the precision rectifier[^30–32^](https://www.zotero.org/google-docs/?TnhgXE).

Indeed, the aforementioned discussion on improving spiking circuit frequency holds true for the retinal pathway, as a reduction of internal capacitance (and thus response time) leads to increased frequency **(Figure S31)**. For example, physiological frequencies can be attained by reducing the internal capacitances of the vOECTs to 5% of their initial value, while maintaining peak currents **(Figure S31)**. In addition, multiple horizontal cells (logic gates) can be used to control the input into the bipolar cell and thus control spiking output **(Figure S32)**. Primarily, the opening of both the devices in the first horizontal cell shunts the effect of the second horizontal cell, similar to the ON-Center OFF-Surround behavior seen in the retina **(Figure S32)**. For example, if the ON detectors are from horizontal cell 2 and the OFF detectors are from horizontal cell 1, then the spiking output only occurs when there is light applied to the ON region and not the off region as seen in biological retinas **(Figure S32)**. Therefore, this work, and the creation of the tunable anti-ambipolar bilayer vOECT, enables the creation of novel retinal pathways with reduced footprint and tunable characteristics as a building block for advanced systems such as artificial retinas, neuroprosthesis, and other intelligent bioelectronic devices[^33,34^](https://www.zotero.org/google-docs/?84YJTa).

References:

[1. Tropp, J., Meli, D. & Rivnay, J. Organic mixed conductors for electrochemical transistors. *Matter* (2023) doi:https://doi.org/10.1016/j.matt.2023.05.001.](https://www.zotero.org/google-docs/?osIEYA)

[2. Harikesh, P. C. *et al.* Ion-tunable antiambipolarity in mixed ion–electron conducting polymers enables biorealistic organic electrochemical neurons. *Nat. Mater.* **22**, 242–248 (2023).](https://www.zotero.org/google-docs/?osIEYA)

[3. Rivnay, J. *et al.* Organic electrochemical transistors. *Nat. Rev. Mater.* **3**, 17086 (2018).](https://www.zotero.org/google-docs/?osIEYA)

[4. Donahue, M. J. *et al.* High-Performance Vertical Organic Electrochemical Transistors. *Adv. Mater.* **30**, 1705031 (2018).](https://www.zotero.org/google-docs/?osIEYA)

[5. Rashid, R. B. *et al.* Ambipolar inverters based on cofacial vertical organic electrochemical transistor pairs for biosignal amplification. *Sci. Adv.* **7**, eabh1055 (2021).](https://www.zotero.org/google-docs/?osIEYA)

[6. Ersman, P. A. *et al.* Screen printed digital circuits based on vertical organic electrochemical transistors. *Flex. Print. Electron.* **2**, 045008 (2017).](https://www.zotero.org/google-docs/?osIEYA)

[7. Huang, H.-M. *et al.* Quasi-Hodgkin–Huxley Neurons with Leaky Integrate-and-Fire Functions Physically Realized with Memristive Devices. *Adv. Mater.* **31**, 1803849 (2019).](https://www.zotero.org/google-docs/?osIEYA)

[8. Beck, M. E. *et al.* Spiking neurons from tunable Gaussian heterojunction transistors. *Nat. Commun.* **11**, 1565 (2020).](https://www.zotero.org/google-docs/?osIEYA)

[9. Harikesh, P. C. *et al.* Organic electrochemical neurons and synapses with ion mediated spiking. *Nat. Commun.* **13**, 901 (2022).](https://www.zotero.org/google-docs/?osIEYA)

[10. Sarkar, T. *et al.* An organic artificial spiking neuron for in situ neuromorphic sensing and biointerfacing. *Nat. Electron.* **5**, 774–783 (2022).](https://www.zotero.org/google-docs/?osIEYA)

[11. Mahowald, M. & Douglas, R. A silicon neuron. *Nature* **354**, 515–518 (1991).](https://www.zotero.org/google-docs/?osIEYA)

[12. Wijekoon, J. H. B. & Dudek, P. Compact silicon neuron circuit with spiking and bursting behaviour. *Adv. Neural Netw. Res. IJCNN ’07* **21**, 524–534 (2008).](https://www.zotero.org/google-docs/?osIEYA)

[13. Indiveri, G. *et al.* Neuromorphic Silicon Neuron Circuits. *Front. Neurosci.* **5**, (2011).](https://www.zotero.org/google-docs/?osIEYA)

[14. Sourikopoulos, I. *et al.* A 4-fJ/Spike Artificial Neuron in 65 nm CMOS Technology. *Front. Neurosci.* **11**, (2017).](https://www.zotero.org/google-docs/?osIEYA)

[15. Yi, W. *et al.* Biological plausibility and stochasticity in scalable VO2 active memristor neurons. *Nat. Commun.* **9**, 4661 (2018).](https://www.zotero.org/google-docs/?osIEYA)

[16. Pickett, M. D., Medeiros-Ribeiro, G. & Williams, R. S. A scalable neuristor built with Mott memristors. *Nat. Mater.* **12**, 114–117 (2013).](https://www.zotero.org/google-docs/?osIEYA)

[17. Hosseini, M. Organic electronics Axon-Hillock neuromorphic circuit: towards biologically compatible, and physically flexible, integrate-and-fire spiking neural networks. *J. Phys. Appl. Phys.* **54**, (2020).](https://www.zotero.org/google-docs/?osIEYA)

[18. *Principles of Neural Science*. (Elsevier, 1991).](https://www.zotero.org/google-docs/?osIEYA)

[19. Lee, Y., Park, H.-L., Kim, Y. & Lee, T.-W. Organic electronic synapses with low energy consumption. *Joule* **5**, 794–810 (2021).](https://www.zotero.org/google-docs/?osIEYA)

[20. Hodgkin, A. L. & Huxley, A. F. A quantitative description of membrane current and its application to conduction and excitation in nerve. *J. Physiol.* **117**, 500–544 (1952).](https://www.zotero.org/google-docs/?osIEYA)

[21. Friedlein, J. T., McLeod, R. R. & Rivnay, J. Device physics of organic electrochemical transistors. *Org. Electron.* **63**, 398–414 (2018).](https://www.zotero.org/google-docs/?osIEYA)

[22. Xu, Y., Gao, S., Li, Z., Yang, R. & Miao, X. Adaptive Hodgkin–Huxley Neuron for Retina-Inspired Perception. *Adv. Intell. Syst.* **4**, 2200210 (2022).](https://www.zotero.org/google-docs/?osIEYA)

[23. Keene, S. T., Rao, A. & Malliaras, G. G. The relationship between ionic-electronic coupling and transport in organic mixed conductors. *Sci. Adv.* **9**, eadi3536.](https://www.zotero.org/google-docs/?osIEYA)

[24. Schuman, C. D. *et al.* Opportunities for neuromorphic computing algorithms and applications. *Nat. Comput. Sci.* **2**, 10–19 (2022).](https://www.zotero.org/google-docs/?osIEYA)

[25. Lim, K. *et al.* The enhancement of electrical and optical properties of PEDOT:PSS using one-step dynamic etching for flexible application. *Org. Electron.* **15**, 1849–1855 (2014).](https://www.zotero.org/google-docs/?osIEYA)

[26. C. Ionescu, P. Svasta, A. Vasile, & D. Bonfert. Investigations on organic printed resistors based on PEDOT:PSS. in *2012 IEEE 18th International Symposium for Design and Technology in Electronic Packaging (SIITME)* 85–89 (2012). doi:10.1109/SIITME.2012.6384352.](https://www.zotero.org/google-docs/?osIEYA)

[27. Fan, X. *et al.* PEDOT:PSS for Flexible and Stretchable Electronics: Modifications, Strategies, and Applications. *Adv. Sci.* **6**, 1900813 (2019).](https://www.zotero.org/google-docs/?osIEYA)

[28. Cucchi, M., Parker, D., Stavrinidou, E., Gkoupidenis, P. & Kleemann, H. In Liquido Computation with Electrochemical Transistors and Mixed Conductors for Intelligent Bioelectronics. *Adv. Mater.* **35**, 2209516 (2023).](https://www.zotero.org/google-docs/?osIEYA)

[29. Ji, X. *et al.* Mimicking associative learning using an ion-trapping non-volatile synaptic organic electrochemical transistor. *Nat. Commun.* **12**, 2480 (2021).](https://www.zotero.org/google-docs/?osIEYA)

[30. Dyson, M. J. *et al.* Color Determination from a Single Broadband Organic Photodiode. *Adv. Opt. Mater.* **8**, 1901722 (2020).](https://www.zotero.org/google-docs/?osIEYA)

[31. Chow, P. C. Y. & Someya, T. Organic Photodetectors for Next-Generation Wearable Electronics. *Adv. Mater.* **32**, 1902045 (2020).](https://www.zotero.org/google-docs/?osIEYA)

[32. Nie, R. *et al.* Highly Sensitive and Broadband Organic Photodetectors with Fast Speed Gain and Large Linear Dynamic Range at Low Forward Bias. *Small* **13**, 1603260 (2017).](https://www.zotero.org/google-docs/?osIEYA)

[33. Guo, X., Xiang, J., Zhang, Y. & Su, Y. Integrated Neuromorphic Photonics: Synapses, Neurons, and Neural Networks. *Adv. Photonics Res.* **2**, 2000212 (2021).](https://www.zotero.org/google-docs/?osIEYA)

[34. van de Burgt, Y., Melianas, A., Keene, S. T., Malliaras, G. & Salleo, A. Organic electronics for neuromorphic computing. *Nat. Electron.* **1**, 386–397 (2018).](https://www.zotero.org/google-docs/?osIEYA)
